# Transcatheter Aortic Valve Implantation in Complex Anatomy: An Anatomy-Driven Framework for Bicuspid Stenosis, Pure Aortic Regurgitation, and the Small Annulus in the Era of Lifetime Management

**DOI:** 10.3390/jcm15197452

**Published:** 2026-09-24

**Authors:** Daniel Miron Brie, Cristian Mornoș, Roxana Popescu, Alina Diduța Brie

**Affiliations:** 1 Cardiovascular Disease Institute Timisoara, Gheorghe Adam Street, No. 13A, 300310 Timisoara, Romania; 2 Research Center, Institute of Cardiovascular Diseases, Gheorghe Adam Street, No. 13A, 300310 Timisoara, Romania; 3 Department of Cardiology, “Victor Babeș” University of Medicine and Pharmacy Timisoara, Eftimie Murgu Square, No. 2, 300041 Timisoara, Romania; 4 Department of Cell and Molecular Biology, “Victor Babeș” University of Medicine and Pharmacy, Tudor Vladimirescu Street, No. 14, 300174 Timisoara, Romania; popescu.roxana@umft.ro (R.P.);; 5 ANAPATMOL Research Center, “Victor Babeș” University of Medicine and Pharmacy, Tudor Vladimirescu Street, No. 14, 300174 Timisoara, Romania; 6 “Louis Țurcanu” Emergency Children Hospital, Doctor Iosif Nemoianu Street, No. 2, 300011 Timisoara, Romania

**Keywords:** transcatheter aortic valve implantation, transcatheter aortic valve replacement, bicuspid aortic valve, aortic stenosis, pure native aortic regurgitation, small aortic annulus, prosthesis–patient mismatch, computed tomography, lifetime management, Heart Team

## Abstract

**Background**: Transcatheter aortic valve implantation is established for severe calcific tricuspid aortic stenosis, but its application in bicuspid aortic valve (BAV) stenosis, pure native aortic regurgitation (AR), and small aortic annulus anatomy remains limited by heterogeneous morphology, device-specific constraints, and uncertain long-term outcomes. These settings require decisions that extend beyond immediate procedural success to encompass coronary access, reintervention, valve durability, and associated aortopathy. This review synthesizes current evidence and proposes an anatomy-driven framework integrating imaging, device selection, procedural planning, and lifetime management. **Methods**: A structured narrative review was conducted in accordance with the Scale for the Assessment of Narrative Review Articles (SANRA) criteria. PubMed/MEDLINE, Embase, and the Cochrane Central Register of Controlled Trials were searched from 1 January 2010 to 31 March 2026 for studies addressing TAVI in BAV stenosis, pure native AR, and small aortic annulus anatomy. Randomized trials, meta-analyses, multicenter registries, pivotal device studies, and guideline or consensus documents were prioritized. After screening 2663 records and assessing 330 full-text reports, 101 records were retained for qualitative synthesis. Evidence was appraised by anatomical substrate, imaging phenotype, device strategy, procedural hazards, and implications for lifetime management. **Results**: In BAV stenosis, contemporary registry and prospective cohort data support high device success and acceptable early and mid-term outcomes in carefully selected patients; however, calcified raphe, bulky asymmetric leaflet or left ventricular outflow tract calcification, annular eccentricity, and concomitant aortopathy identify higher-risk phenotypes that often favor surgery. Randomized evidence in younger low-risk BAV patients remains limited and exploratory, with the NOTION-2 trial showing a numerically higher 3-year composite event rate after TAVI than surgical aortic valve replacement (20.4% vs. 7.8%; hazard ratio 2.9, 95% confidence interval 0.9–9.0) in the small bicuspid subgroup. In pure native AR, lack of annular calcification and frequent root dilatation impair anchoring of conventional transcatheter heart valves, increasing risks of migration, embolization, residual AR, and second-valve implantation. Dedicated leaflet-anchoring devices have improved outcomes: in ALIGN-AR, the JenaValve Trilogy system achieved procedural success above 95% in high-surgical-risk patients, although evidence remains single-arm and nonrandomized. In small annuli, the strongest comparative evidence comes from the SMART trial, in which supra-annular self-expanding valves were non-inferior to balloon-expandable valves for the 12-month clinical composite and reduced moderate/severe prosthesis–patient mismatch at 30 days (11.2% vs. 35.3%). Across all three phenotypes, electrocardiography-gated computed tomography is fundamental for defining anchoring, sizing, calcium distribution, root and coronary anatomy, anticipated coronary re-access, and the feasibility of redo-TAVI. Surgery remains particularly relevant when aortic repair, annular/root enlargement, or a more durable lifetime strategy is required. **Conclusions**: TAVI is feasible in selected patients with BAV stenosis, pure native AR, and small aortic annuli, but the evidentiary strength and optimal procedural strategy differ substantially across these anatomies. Treatment should be guided by an anatomy-driven, computed-tomography-based Heart Team assessment that integrates immediate procedural feasibility with age, surgical candidacy, aortopathy, prosthesis–patient mismatch risk, coronary access, durability, and future reintervention options. Surgery remains the more established strategy for many younger, operable patients with unfavorable BAV morphology or aortopathy, AR with root dilatation, or very small annuli amenable to enlargement. Dedicated randomized trials, standardized imaging criteria, and long-term durability data are needed to refine lifetime management in complex aortic valve anatomy.

## 1. Background

Transcatheter aortic valve implantation has evolved over two decades from a bail-out therapy for inoperable patients to an established treatment for severe aortic stenosis across the surgical risk spectrum, supported by multiple randomized trials and long-term follow-up showing non-inferiority or superiority to surgical aortic valve replacement in high-, intermediate-, and selected low-risk patients [1]. In much of the developed world, TAVI is now the default strategy for older patients with tricuspid aortic stenosis, and the contemporary ESC/EACTS guideline [2] endorses earlier intervention and CT-based planning within Heart Valve Centers. However, the randomized evidence establishing TAVI was based on a constrained anatomical substrate. Patients with bicuspid aortic valve (BAV) stenosis were excluded from pivotal low-risk trials, pure native aortic regurgitation (AR) lay outside the labeled indication, and small-annulus patients were enrolled but rarely analyzed as a prespecified subgroup.

Existing reviews typically address one anatomy in isolation or focus on procedural technique; what is lacking is an anatomy-driven synthesis that links CT phenotype to device choice and to lifetime management across bicuspid stenosis, pure regurgitation, and the small annulus. Similarly, while dedicated analyses and systematic reviews indicate that TAVI can provide acceptable hemodynamics in small annuli, device selection remains debated, and no consensus exists on the optimal strategy, underscoring a substantial evidence gap and the need for further randomized studies in these anatomically complex populations. As TAVI is offered to younger patients with longer life expectancy, these three “complex” anatomies are encountered with increasing frequency, and the evidence guiding their management is correspondingly fragmented [3]. Device durability, the feasibility of future coronary access and redo procedures, and—in BAV—the natural history of concomitant aortopathy therefore become first-order considerations rather than afterthoughts [4]. Most existing reviews address a single anatomy in isolation or focus narrowly on procedural technique; what remains lacking is a synthesis that explicitly links CT phenotype to device choice and to lifetime management across all three anatomies. In particular, the existing literature is fragmented by anatomy, limited by non-standardized CT framing, and insufficiently connected to a coherent lifetime strategy—the specific gap this review seeks to address. This narrative review has three aims: (i) to summarize current evidence for TAVI in BAV stenosis, pure AR, and the small annulus, incorporating the most recent randomized and registry data and appraising its quality explicitly; (ii) to propose a practical, anatomy-driven decision framework that couples imaging findings to device selection, implantation strategy, and lifetime management; and (iii) to define, for each anatomy, a structured research agenda that moves beyond the generic call for “more data”.

Unlike prior reviews that largely compile observational data and conclude with a generic request for more evidence, this work explicitly grades the strength and limitations of both recent randomized trials and registry experience, and then translates those data into concrete, imaging-based decision pathways linking morphology (BAV subtype, root and annular dimensions, calcification pattern, leaflet and aortopathy characteristics) to specific device choices, implantation techniques, and lifetime management considerations.

The certainty and maturity of evidence differ substantially across the three anatomical settings. Evidence in bicuspid aortic stenosis is derived predominantly from registries, selected prospective cohorts, and limited randomized subgroup data; evidence in pure native aortic regurgitation remains largely single-arm, device-specific, and observational; and the small-annulus setting has comparatively stronger randomized evidence for hemodynamic endpoints, although long-term clinical and lifetime management outcomes remain incompletely defined. Accordingly, this framework is intended to structure interpretation and Heart Team discussion, not to provide a validated treatment algorithm or replace guideline-directed, individualized decision-making.

## 2. Methods

This structured narrative review follows the SANRA (Scale for the Assessment of Narrative Review Articles) quality criteria and integrates heterogeneous evidence (randomized trials, registries, device-specific studies, and guidelines) into a clinically usable framework, rather than generating pooled effect estimates. To improve methodological transparency and support editorial verification, we provide a SANRA adherence checklist for this narrative review in Supplementary Table S1. We searched PubMed/MEDLINE, Embase, and the Cochrane Central Register of Controlled Trials from 1 January 2010 to 31 March 2026. The search combined three concept blocks with Boolean operators: (i) the intervention (“transcatheter aortic valve” OR TAVI OR TAVR); (ii) the anatomy (“bicuspid aortic valve” OR “aortic regurgitation” OR “aortic insufficiency” OR “small aortic annulus” OR “prosthesis-patient mismatch”); and (iii) outcome and planning terms (“computed tomography” OR sizing OR durability OR “coronary access” OR “commissural alignment” OR redo). We additionally hand-searched the reference lists of key articles, relevant consensus documents, and the 2025 ESC/EACTS guidelines, and screened late-breaking presentations from major cardiology congresses through the first quarter of 2026. Database searches returned 4575 records (PubMed/MEDLINE, n = 1842; Embase, n = 2415; Cochrane CENTRAL, n = 318), and an additional 61 records were identified through citation searching, guideline and consensus documents, and congress late-breaking presentations. After removal of 1463 duplicates and 449 records excluded before screening on language or publication type, 2663 records were screened by title and abstract, and 330 full-text reports were assessed for eligibility. We excluded 229 reports at full text, most commonly because the anatomy did not correspond to one of the three target phenotypes, because the population overlapped with a report retained in preference, or because the record was a case report or a congress abstract without subsequent peer-reviewed publication. We retained 101 records for synthesis; three antedate the search window and were identified by citation searching as foundational classification or definition papers. Supplementary Materials S2 provides the complete database-specific yields, deduplication details, reasons for exclusion at full text, and the corresponding PRISMA 2020-style flow diagram.

Titles/abstracts and then full texts were screened for English-language randomized controlled trials, meta-analyses, multicenter registries, pivotal device studies, and guideline or consensus documents. Case reports, conference abstracts without subsequent peer-reviewed publication (except where they represented the only data on practice-changing cohorts and are identified as such), and non-English articles were excluded. When multiple publications described overlapping populations, we prioritized randomized trials, then propensity-matched or core-lab-adjudicated registries, followed by unmatched series and single-arm device studies, retaining the most recent report with the most complete follow-up. Given the heterogeneity of designs and anatomy definitions, we synthesized findings qualitatively by anatomy and flagged major design limitations; we did not perform formal risk-of-bias scoring or quantitative pooling, consistent with the narrative design. A SANRA adherence checklist is provided in Supplementary Table S1. Because this was a structured narrative review rather than a systematic review, we report the search sources, dates, eligibility criteria, and evidence-prioritization principles; we provide exact screening counts only where verifiable from the search records. We additionally hand-searched the reference lists of key peer-reviewed articles, formal guideline and consensus documents, and relevant regulatory sources. We considered conference presentations and other non-peer-reviewed materials only when they reported potentially practice-relevant contemporary data not yet available in full publication; we explicitly identified such evidence as preliminary and did not use it as the sole basis for definitive clinical recommendations. We additionally hand-searched the reference lists of key articles, relevant consensus documents, and the 2025 ESC/EACTS guidelines, and screened late-breaking presentations from major cardiology congresses through the first quarter of 2026. Literature searches were conducted using PubMed/MEDLINE (National Library of Medicine, Bethesda, MD, USA; https://pubmed.ncbi.nlm.nih.gov/; accessed on 31 March 2026), Embase (Elsevier B.V., Amsterdam, The Netherlands; https://www.embase.com/; accessed on 31 March 2026), and the Cochrane Central Register of Controlled Trials (Cochrane, London, UK; https://www.cochranelibrary.com/central; accessed on 31 March 2026). No dedicated statistical-analysis software was used because this was a structured narrative review without quantitative pooling or meta-analysis.

## 3. Specific Complex Anatomy

### 3.1. Bicuspid Aortic Valve Stenosis

BAV is the most common congenital cardiac malformation, affecting approximately 1–2% of the population, and accounts for a substantial and rising proportion of TAVI candidates [5,6]. The Sievers classification stratifies BAV by the number and spatial arrangement of raphes into type 0 (no raphe), type 1 (one raphe, most common), and type 2 (two raphes), the last overlapping phenotypically with unicuspid valves [7]; an international consensus statement has since refined this nomenclature for the imaging era [6]. Relative to tricuspid AS, bicuspid anatomy is characterized by an elliptical and often larger annulus, eccentric and bulky leaflet calcification, frequent calcification of the raphe, and a supra-annular constraint at the level of the fused commissures. Patients with BAV develop severe AS roughly a decade earlier than those with tricuspid valves and frequently harbor concomitant ascending aortopathy, which TAVI does not address and whose natural history continues after valve replacement [8,9]. These features increase the risk of malposition, paravalvular regurgitation, and device under- or oversizing, and may predispose to annular or root injury when aggressive oversizing is used to secure the valve. In addition, concomitant ascending aortopathy and root dilatation, frequent in BAV disease, complicate the definition of an appropriate landing zone and raise lifetime management issues that are difficult to address with a single transcatheter intervention. TAVI in BAV stenosis remains a technically more demanding procedure, requiring meticulous CT-based phenotyping and individualized strategy, and key questions about long-term durability and optimal patient selection are still incompletely answered [10]. The coexistence of a calcified raphe and excess leaflet calcification constitutes a particularly unfavorable bicuspid phenotype for TAVI. This CT pattern may constrain transcatheter heart valve expansion, impair sealing, and concentrate radial forces at the annular and aortic root level. Registry data associate this combined phenotype with higher risks of moderate/severe paravalvular leak, aortic root injury, and mortality than bicuspid anatomy without these features. Therefore, CT evaluation should report not only the overall calcium burden but also the presence and severity of raphe calcification, asymmetry and distribution of leaflet calcium, annular eccentricity, and associated left ventricular outflow tract (LVOT) calcium. In younger, operable patients—especially when concomitant aortopathy is present—this phenotype generally favors surgery; if TAVI is pursued, conservative sizing and cautious use of balloon dilatation are essential.

### 3.2. Pure Aortic Regurgitation

Pure native AR arises from primary leaflet disease, aortic root or annular dilation, or a combination of the two. Pure native AR should be classified according to its dominant anatomical mechanism: primary leaflet pathology versus functional AR caused by annular, aortic root, or ascending aortic dilatation with secondary malcoaptation. Leaflet-predominant AR may retain a more definable landing and anchoring zone and may be considered for TAVI with a dedicated device in highly selected patients who are ineligible for surgery, whereas root- or ascending-aorta-driven AR is frequently associated with a large, dynamic, non-calcified annulus and untreated aortopathy, making transcatheter treatment less secure and often strategically incomplete. Accordingly, CT-based evaluation of leaflet morphology, coaptation, annular dimensions, root/ascending aortic anatomy, and the need for concomitant aortic repair should guide device selection and the choice between TAVI and surgery [2,11]. Pure native AR creates a fundamentally different, more hostile environment for current TAVI devices than calcific aortic stenosis does, primarily because most prostheses were designed for rigid, calcified anatomy. In chronic AR, the aortic root and annulus are often dilated, the leaflets are mobile and non-calcified, and systolic and diastolic flow generate highly dynamic forces within the root, all of which reduce frictional fixation and increase the risk of valve migration, embolization, and significant residual regurgitation after deployment. The absence of circumferential calcium and frequent annular enlargement make standard “oversizing” strategies less reliable and may require extreme oversizing or dedicated devices with active anchoring elements, both of which can carry additional procedural risk. Moreover, patients with pure AR exhibit markedly heterogeneous root and LVOT anatomies compared with those in typical AS populations, making device behavior less predictable and the learning curve steeper, particularly for centers with limited experience. Historically, these factors led to pure AR being regarded as a relative contraindication for TAVI, and most evidence still comes from off-label use, early-generation valves, and small registries, although newer dedicated and repositionable systems are beginning to show improved device success and lower rates of moderate–severe residual AR. Consequently, TAVI in pure AR remains a niche, high-complexity indication where meticulous anatomical assessment and careful patient selection are critical, and robust randomized data are still lacking [11,12]. In patients with aortic regurgitation, bicuspid TAVI may be procedurally feasible yet strategically incomplete when concomitant aortopathy is present, because correction of the regurgitant valve lesion alone does not treat the diseased aortic root or ascending aorta that may continue to drive long-term risk. This limitation is especially relevant in bicuspid AR, where durable management often requires an integrated valve–aortic strategy rather than an isolated transcatheter solution, particularly when aortic dimensions already meet or approach thresholds for surgical repair.

### 3.3. Small Aortic Annulus

Definitions of a small aortic annulus vary across studies and device platforms. An annular area <430 mm^2^, corresponding to the eligibility threshold used in the SMART trial, is a widely used research definition for a small annulus. A more restrictive threshold of approximately <400 mm^2^ may be used pragmatically to identify a particularly small annular subgroup in whom the risks of elevated residual gradients, PPM, limited device options, and future valve-in-valve constraints may be especially relevant. These thresholds should not be considered interchangeable or used as isolated treatment cut-offs. In clinical practice, annular area should be interpreted together with perimeter, LVOT geometry, predicted indexed effective orifice area, body size, coronary anatomy, calcification, and device-specific sizing recommendations [13,14]. Small annuli are disproportionately common in elderly women, and coexisting conditions, such as hypertrophic remodeling of the left ventricular outflow tract, can further constrain the achievable effective orifice area [15]. A small aortic annulus represents a prototypical “high-stakes” anatomy in TAVI because the limited annular area constrains prosthesis size and predisposes to high residual gradients and PPM, with direct implications for symptoms and long-term outcomes. Patients with small annuli often have smaller body size but relatively high stroke volume demands, so even modestly elevated post-TAVI gradients or a reduced effective orifice area can translate into clinically relevant functional limitation and potentially impaired survival. From a technical standpoint, annular undersizing increases the risk of device underexpansion and PPM, whereas aggressive oversizing to improve hemodynamics raises the likelihood of annular rupture, coronary obstruction, or conduction disturbances, making sizing decisions particularly delicate. Comparative studies and registries indicate that self-expanding supra-annular valves generally provide larger effective orifice areas and lower gradients in small annuli than intra-annular balloon-expandable or mechanically expanding valves, but at the cost of a higher incidence of paravalvular regurgitation. Despite encouraging short-term results showing good hemodynamics and low rates of severe PPM in selected cohorts, data remain limited for “extra-small” annuli, and for younger, low-risk patients, so optimal device design, implantation strategy, and long-term durability in this anatomy remain active areas of investigation [13,16,17].

Across all three anatomies, “complexity” reduces to a common set of mechanical problems—secure anchoring, accurate sizing, and avoiding annular rupture, coronary obstruction, and PPM—that manifest differently depending on the substrate. Anatomically, each of these problems maps onto a characteristic procedural failure mode: under-expansion and paravalvular leak in the calcium-eccentric bicuspid valve, migration and embolization in the calcium-poor regurgitant root, and high residual gradients and impaired leaflet washout in the small annulus, with conduction disturbance and compromised future coronary access cutting across all three [16,18]. Table 1 summarizes these anatomy-specific challenges together with the corresponding imaging priorities, device considerations, principal procedural hazards, and evidence gaps.

While this review focuses on an anatomy-driven TAVI strategy, lifetime management must explicitly consider surgical options at baseline and at reintervention. In bicuspid stenosis with concomitant aortopathy and in pure AR with root dilation, surgery is more “future-proof” because it permits concomitant repair of the root and ascending aorta and enables implantation of a durable surgical bioprosthesis or mechanical valve. Surgical aortic valve replacement offers several annular/root enlargement techniques—classically Nicks and Manouguian, and more recently the Y-incision/Yang technique—that allow implantation of a larger prosthesis and mitigate PPM [19,20]. Also, rapid-deployment/sutureless bioprostheses that can mitigate PPM and facilitate subsequent valve-in-valve procedures can be a solution. A coherent lifetime management framework therefore requires, for each anatomy, a structured comparison between contemporary TAVI strategies and surgical approaches, balancing early procedural risk against durability, reintervention options, and coronary access over decades rather than years.

## 4. Imaging and Procedural Planning

Contrast-enhanced, ECG-gated multidetector CT is the cornerstone of planning across all three anatomies, providing annular dimensions, leaflet morphology, and calcium distribution, coronary ostial heights, sinus and sinotubular junction geometry, and access-vessel assessment [21,22]. Standardized calcium quantification refines both sizing and the anticipated risk of paravalvular leak, and the 2025 guidelines also position coronary CT angiography as the first-line test to exclude significant coronary disease in patients with a low-to-intermediate pre-test likelihood [2]. However, the imaging task differs by substrate, and a structured checklist (Table 2) helps ensure anatomy-specific measurements are captured prospectively rather than reconstructed after a complication.

### 4.1. Bicuspid Aortic Stenosis

Imaging and procedural planning are central to successful TAVI in BAV stenosis because anatomy is highly heterogeneous and more complex than in tricuspid aortic stenosis. Multislice CT is the cornerstone, allowing detailed assessment of BAV subtype (e.g., Sievers classification), raphe presence, leaflet asymmetry, annular ellipticity, calcium distribution, and associated aortopathy, all of which influence the device sizing, choice, and implantation strategy. CT-derived measurements should integrate annular, supra-annular, and LVOT dimensions, with particular attention to eccentricity and tapered roots, to avoid both undersizing (risk of paravalvular leak and migration) and oversizing (risk of annular or root injury) [23]. 3D reconstruction and fusion imaging can help define optimal fluoroscopic projection angles and anticipate challenging deployment scenarios such as heavy raphe calcification or asymmetric cusp opening. Echocardiography remains essential for quantifying severity, assessing concomitant regurgitation, and evaluating ventricular and aortic root function, complementing CT in borderline anatomical cases. Procedurally, many centers favor self-expanding or supra-annular valves in selected BAV anatomies to maximize effective orifice area, but device selection is increasingly individualized based on CT morphology rather than on a single preferred platform. Strategies such as higher or lower implantation, careful pre- and post-dilatation, and consideration of cerebral embolic protection are tailored to the degree and location of calcification and the risk of conduction disturbances or stroke. Given the persistent risks of paravalvular leak, pacemaker implantation, and annular complications in BAV, Heart Team discussion that integrates multimodality imaging, lifetime management, and surgical options remains mandatory during planning [24].

### 4.2. Pure AR

In pure AR, sizing is performed off the annulus with generally more aggressive oversizing when a non-dedicated device is used, and planning must explicitly estimate the anchoring zone across the annulus, left ventricular outflow tract, and sinotubular junction, recognizing the elevated migration risk; dedicated devices carry their own sizing charts and engage the native leaflets directly. Intraprocedural indices such as the aortic regurgitation index have been used to gauge residual regurgitation, but their applicability to the dynamic AR annulus is limited.

Imaging and procedural planning for TAVI in pure or predominant native AR are uniquely demanding because operators must compensate for the lack of calcific anchoring and frequently dilated aortic root anatomy. Multislice CT is central to case selection, providing accurate measurements of annular, LVOT, and root dimensions, assessment of cusp morphology and coaptation level, and detection of root or ascending aorta dilatation that may preclude a stable landing zone for a transcatheter valve. CT and echocardiography together help distinguish focal leaflet disease from more diffuse root pathology, quantify regurgitant severity and LV remodeling, and identify patients in whom TAVI is being considered only because surgical risk is prohibitive. Because conventional TAVI devices were optimized for calcific stenosis, AR planning emphasizes selecting self-expanding or dedicated valves with active anchoring mechanisms and significant radial force, often accepting some degree of oversizing to improve fixation while carefully balancing the risks of annular injury and conduction disturbances. Procedurally, operators may favor transapical or alternative access in selected anatomies to gain more tactile feedback and control during deployment, and often adopt stepwise release, rapid pacing, and intra-procedural TOE or fusion imaging to minimize valve migration and residual regurgitation. Given that most experience with native AR comes from small registries, off-label use, and early cohorts with high device failure rates, Heart Team deliberation that integrates imaging findings, surgical options, and evolving guideline recommendations remains essential before offering TAVI as a treatment strategy [25].

### 4.3. Small Annulus

For procedural planning, the CT-derived annular area is interpreted as a continuous measurement rather than through a single universal threshold. An area <430 mm^2^ identifies the broader trial-defined small-annulus population, whereas an area <400 mm^2^ may flag a more extreme anatomical subgroup requiring particular attention to predicted prosthesis–patient mismatch, residual gradients, annular injury risk, coronary obstruction, and future reintervention options. Device selection and sizing should therefore integrate annular area with the perimeter, diameters, LVOT dimensions, leaflet and LVOT calcification, body surface area, predicted indexed effective orifice area, coronary height, sinus dimensions, and manufacturer-specific sizing charts. In this anatomy, small errors in sizing can translate into either high residual gradients and PPM (when undersized) or annular rupture and coronary obstruction (when aggressively oversized), so sizing algorithms typically integrate multiple CT measurements and device-specific charts rather than relying on diameter alone. Echocardiography complements CT by confirming stenosis severity, assessing ventricular function, and estimating body surface area, which is essential for indexing effective orifice area and anticipating the risk of PPM in smaller patients, particularly women. From a device strategy perspective, self-expanding supra-annular valves are often favored in very small annuli because they can provide larger effective orifice areas and lower gradients, but this choice is weighed against a higher propensity for paravalvular regurgitation. Procedural planning also incorporates coronary height, sinus size, and leaflet calcification to anticipate coronary obstruction or annular injury, with pre-emptive techniques such as BASILICA, coronary protection, or surgical backup considered in selected high-risk cases. Overall, a structured, CT-driven workflow that links anatomical findings to specific device selection, implantation depth, and bailout strategies is critical to safely expanding TAVI indications in patients with small annuli [26].

## 5. TAVI in Bicuspid Aortic Stenosis

Observational and registry data have consistently shown that contemporary THVs achieve high device success and acceptable short- and mid-term outcomes in selected BAV patients, although these datasets share the recurring limitations of nonrandomized comparisons: residual confounding, the selection of anatomically favorable candidates, and treatment at high-volume expert centers. In an early large analysis from the STS/ACC TVT Registry, propensity-matched bicuspid and tricuspid patients had comparable mortality and stroke, albeit with slightly higher rates of paravalvular regurgitation in the bicuspid group [27]. Subsequent analyses of low-surgical-risk patients treated with a contemporary balloon-expandable valve reproduced this pattern, with high procedural success and 30-day and 1-year death and stroke rates that did not differ significantly from tricuspid controls [28]. Analogous registry data with the self-expanding Evolut platform likewise showed comparable 30-day and 1-year mortality and stroke [29], and dedicated feasibility and study cohorts—the Low Risk Bicuspid TAVR study, the Evolut Low Risk Bicuspid Study, and the PARTNER 3 bicuspid registry—reported reassuring early safety in carefully selected patients [30,31,32]. Larger comparative and temporal trend analyses have broadly supported the approach’s feasibility while underscoring its dependence on morphology and calcium burden [33,34,35]. Medium-term follow-up from the Evolut Low Risk Bicuspid Study shows low rates of death or disabling stroke and sustained hemodynamic performance out to three years, without moderate or severe paravalvular regurgitation, albeit with relatively frequent new pacemaker implantation. These data are reassuring but do not address true long-term durability in patients who may outlive one or more bioprostheses, underscoring the need for caution when extrapolating TAVI into younger bicuspid populations.

We agree that the original wording overstated the certainty of the comparative evidence for TAVI versus SAVR in younger, low-risk patients with bicuspid aortic stenosis. Although the NOTION-2 3-year analysis showed a numerically higher composite event rate after TAVI in the bicuspid subgroup (20.4% versus 7.8%; hazard ratio 2.9, 95% CI 0.9–9.0), this subgroup was small, and the comparison was not statistically significant; it should therefore be regarded as exploratory rather than definitive evidence of surgical superiority. In the overall NOTION-2 population, 3-year outcomes were similar between TAVI and SAVR, and moderate-or-greater structural valve deterioration was infrequent and comparable between treatment groups, providing no basis for firm conclusions about long-term comparative durability in bicuspid anatomy. Accordingly, the choice between TAVI and SAVR in younger, low-risk bicuspid patients should be individualized through multidisciplinary Heart Team assessment, integrating valve morphology, calcified raphe and leaflet calcium burden, annular and supra-annular geometry, associated aortopathy, coronary anatomy, procedural feasibility, life expectancy, and anticipated reintervention options. Surgery remains particularly compelling when concomitant aortic repair is indicated or when anatomy is unfavorable for transcatheter treatment, whereas TAVI may be considered in carefully selected anatomically suitable patients at experienced Heart Valve Centers. This approach is consistent with current guidance emphasizing clinical, anatomical, procedural, and lifetime management factors rather than a universal procedure-first strategy.

The randomized evidence is more nuanced and, critically, more limited. NOTION-2 [36] is the first randomized trial to enroll low-risk patients (aged ≤ 75 years) with either tricuspid or bicuspid AS and to compare TAVI with surgery. Overall, the 1-year composite of death, stroke, or rehospitalization was similar between arms; however, an important interaction emerged by valve morphology, with less favorable outcomes after TAVI in bicuspid patients. At three years, the signal persisted: outcomes remained similar in tricuspid AS, whereas bicuspid patients had a numerically higher event rate with TAVI (20.4% versus 7.8%; hazard ratio 2.9; 95% CI 0.9–9.0), a difference that did not reach statistical significance in a modestly sized subgroup, and structural valve deterioration and bioprosthetic valve failure were low and comparable overall [37]. These findings must be interpreted in light of the trial’s limitations: the bicuspid subgroup was small and underpowered; anatomical suitability was not centrally adjudicated; some enrolled “bicuspid” valves were Sievers type 2 morphologies more properly classified as unicuspid; and surgical outcomes reflected experienced centers. They are therefore best read as a call for caution—not a prohibition—when offering TAVI to young bicuspid patients [38]. In the overall NOTION-2 population of low-surgical-risk patients aged 60–75 years, the 3-year composite of death, stroke, or procedure-, valve-, or heart-failure-related hospitalization was similar after TAVI and SAVR. In the bicuspid subgroup, outcome estimates were imprecise because of a limited sample size; therefore, subgroup findings should be considered exploratory rather than definitive evidence of comparative superiority. For younger, operable patients with bicuspid AS, treatment selection should remain individualized through Heart Team assessment of valve morphology, raphe and leaflet calcification, annular and supra-annular dimensions, associated aortopathy, coronary anatomy, anticipated durability, and future reintervention options. Accordingly, NOTION-2 neither proves inferiority of TAVI nor establishes equipoise with surgery in low-risk bicuspid aortic stenosis. Rather, it reinforces the need for cautious patient selection and supports surgery as the more established default strategy in younger or otherwise operable patients with unfavorable bicuspid morphology, extensive calcification, significant aortopathy, or an anticipated need for concomitant aortic repair.

### 5.1. Favorable and Unfavorable Bicuspid Phenotypes

Not all bicuspid valves behave alike, and the distinction matters more than the binary bicuspid-versus-tricuspid label. Relatively favorable features for TAVI include a symmetric type 1 phenotype without a heavily calcified raphe, a modest and evenly distributed calcium burden, limited annular eccentricity, and a non-dilated ascending aorta and root. Relatively unfavorable features include a calcified raphe, bulky and asymmetric leaflet calcium, marked annular eccentricity, and coexisting aortopathy—each associated with under-expansion, paravalvular leak, or a residual surgical indication for the aorta [27,39]. Device and technique factors modulate these outcomes: self-expanding, repositionable, supra-annular valves may be advantageous in very asymmetric or heavily calcified anatomy, whereas balloon-expandable valves offer predictable deployment, and judicious pre- and post-dilation, attention to implantation depth, and commissural alignment (Section 8) all influence hemodynamics, conduction, and future coronary access [40].

The principal residual uncertainties are the exclusion of BAV from the pivotal low-risk stenosis trials, the paucity of durability data beyond five years in a population that will live for decades, the untreated trajectory of concomitant aortopathy, and the absence of a standardized, validated CT sizing algorithm for bicuspid anatomy. For younger, operable patients—particularly those with a calcified raphe, significant aortopathy, or an anatomy poorly suited to a transcatheter device—surgery remains the more established option [2,9]. In practice, low-risk bicuspid patients with symmetric type 1 morphology, non-dilated aorta, and modest, evenly distributed calcium are reasonable candidates for TAVI at experienced centers, whereas those with a calcified raphe, marked annular eccentricity, bulky asymmetric calcium, or significant ascending aortopathy are better served by surgery, often with concomitant aortic repair. When TAVI is pursued in anatomically borderline cases, strategies such as supra-annular self-expanding valves, careful commissural alignment, and liberal use of cerebral embolic protection may partially mitigate the excess risk observed in randomized trials. In patients with bicuspid anatomy and significant aortopathy, TAVI may be procedurally feasible but strategically incomplete because successful valve implantation does not resolve the concomitant aortopathy, which may remain the dominant determinant of long-term risk and future intervention. Thus, even when transcatheter treatment appears technically achievable, the preferred strategy should be guided by whether the chosen approach addresses the full valvulo-aortic disease process rather than the valve lesion alone.

Table 3 summarizes phenotype-specific considerations intended to support, rather than determine, multidisciplinary treatment selection.

#### Take-Home Message

Taken together, contemporary registry and feasibility data support TAVI as a safe, feasible option for anatomically favorable bicuspid stenosis, with high device success and early outcomes that broadly resemble those in tricuspid disease when performed in expert centers. However, randomized evidence in low-risk patients remains limited and more guarded, with NOTION-2 suggesting that younger bicuspid patients—particularly those with complex morphology—may experience less favorable outcomes with TAVI than with surgery, even if absolute event rates are low. In this setting, valve morphology, calcium distribution, annular eccentricity, and aortic dimensions—not the bicuspid label alone—should drive the choice between TAVI and surgery. By contrast, in older or higher-risk patients with symmetric type 1 morphology, modest and evenly distributed calcium, and a non-dilated ascending aorta, TAVI using contemporary devices can be considered a reasonable alternative within a Heart Team framework, provided that procedural planning (including commissural alignment and consideration of cerebral protection) is meticulous. Ultimately, for bicuspid aortic stenosis, the question is not whether TAVI is possible, but in whom it is truly appropriate, and current data favor a cautious, anatomy-driven, surgery-first posture in young low-risk patients while allowing selective expansion of TAVI in more favorable phenotypes. However, interpret this recommendation cautiously. In younger, low-risk bicuspid patients, robust data on valve durability beyond 5–10 years and on the long-term trajectory of concomitant aortopathy are still lacking, and the favorable outcomes reported to date largely derive from expert, high-volume centers. In younger, low-risk patients with bicuspid AS, the relative merits of TAVI and SAVR should be determined through individualized Heart Team and shared decision-making. Surgery is particularly attractive when aortopathy requires concomitant repair or when leaflet, raphe, annular, coronary, or lifetime management features are unfavorable for TAVI; conversely, TAVI may be reasonable in selected anatomically suitable patients treated at experienced centers.

## 6. TAVI in Pure Aortic Regurgitation

Conventional transcatheter heart valves designed and labeled for calcific aortic stenosis have generally been used off-label in pure native aortic regurgitation. In contrast, the JenaValve Trilogy system is a dedicated leaflet-clasping device designed to anchor independently of annular calcification. It has CE-mark authorization in Europe for symptomatic severe native aortic regurgitation. Regulatory authorization outside Europe is jurisdiction- and time-specific and should be verified locally. In an international 40-center registry of 331 patients, overall device success improved substantially with newer-generation devices (81.1%) compared with early-generation devices (61.3%), driven largely by lower rates of second-valve implantation, but residual regurgitation and migration remained concerns [48]. A systematic review of pooled cohorts reported device success ranging from 74% to 100%, a second valve required in about 7%, 30-day all-cause mortality of approximately 7%, and ≥moderate residual AR in roughly 9% [12]; further multicenter series confirmed the same trade-offs [11,49]. A contemporary multicenter project (PANTHEON) [50] reiterated that, even with newer devices used off-label, residual AR and the need for a second valve remain more frequent than in calcific AS. Early self-expanding and repositionable off-label experiences illustrated both the feasibility and the ceiling of the non-dedicated approach [51,52].

Researchers developed purpose-built devices to directly address anchoring. The JenaValve Trilogy system uses leaflet-clasping “locators” to engage the native cusps and secure the prosthesis without calcium, while its open-cell nitinol frame is designed to preserve coronary access. In the ALIGN-AR pivotal investigational device exemption trial of 180 high-surgical-risk patients with symptomatic severe AR, the Trilogy valve achieved a procedural success rate above 95% and met its pre-specified non-inferiority thresholds for safety and efficacy, with excellent hemodynamics and low paravalvular leak rates maintained through two years [53]. Having held CE mark approval in Europe since 2021, the Trilogy system received FDA approval in the United States in 2026, becoming the first transcatheter heart valve approved specifically for aortic regurgitation [54,55]. FDA PMA P250024 was approved on 17 March 2026. The indication is not all pure AR: it is symptomatic, severe native tricuspid AR, excluding acute endocarditis, rheumatic heart disease, and acute aortic dissection, in patients judged by a Heart Team to be at high or greater surgical risk; “high” includes predicted 30-day surgical mortality ≥8% with relevant comorbidity considerations [56]. This is a jurisdiction-specific regulatory indication and should not be interpreted as evidence of comparative superiority over surgery, nor extrapolated to lower-risk patients, non-tricuspid valve morphology, acute AR, rheumatic disease, or acute aortic dissection.

A second dedicated device, the J-Valve (which also employs a leaflet-grasping mechanism), has now accrued long-term follow-up data in aortic stenosis and regurgitation and is under further prospective investigation, with the JOURNEY trial enrolling [57,58]. A meta-analysis of 2162 patients from 34 studies comparing dedicated with off-label devices in native AR found lower 30-day mortality (3% vs. 9%) and higher device success (90% vs. 82%) with dedicated platforms, though it inherited the observational, single-arm nature of its constituent studies [59]. Indications in pure native aortic regurgitation are highlighted in Table 4.

The 2025 ESC/EACTS guidelines reflect this maturation cautiously, introducing a Class IIb recommendation for TAVI as a treatment option at experienced centers for selected patients with severe AR who are ineligible for surgery. We agree that the evidence base for TAVI in pure native [2]. AR remains limited and should be interpreted cautiously. Current data primarily derive from single-arm pivotal studies, registries, and selected high-risk or inoperable populations treated at experienced centers; therefore, they support technical feasibility in carefully selected patients rather than established equivalence or superiority to SAVR. The 2025 ESC/EACTS Guidelines state that TAVI may be considered at experienced centers for symptomatic patients with severe AR who are ineligible for surgery and have suitable anatomy, whereas surgery remains the standard treatment for operable patients. Dedicated AR devices may improve anchoring and reduce residual regurgitation compared with historical off-label use of AS-designed platforms, but available comparisons are nonrandomized and do not establish superiority over surgery. Regulatory approval and device availability remain jurisdiction-specific; for example, US FDA approval of the Trilogy system applies to symptomatic severe native tricuspid AR in patients at high or greater surgical risk, not to all AR phenotypes or lower-risk populations. Accordingly, TAVI for pure native AR should not be generalized to younger or lower-risk patients and should be considered only after detailed CT-based anatomical assessment and multidisciplinary Heart Team evaluation.

Substantial knowledge gaps nonetheless remain: no randomized comparison of TAVI versus surgery or of dedicated versus non-dedicated devices; device availability is geographically heterogeneous; the optimal definition of the anchoring zone is unsettled; and long-term durability and the criteria for selecting a transcatheter approach surgical approach in operable patients are undefined. Enthusiasm for dedicated devices should therefore be tempered by the reality that the entire evidence base remains nonrandomized and concentrated in a small number of expert centers.

### 6.1. Take-Home Message

TAVI for native aortic regurgitation remains a niche, high-complexity therapy. Conventional AS-designed transcatheter valves are generally used off-label in this setting, whereas dedicated AR devices have region-specific authorization. The available evidence is predominantly single-arm and observational; therefore, treatment should be individualized in experienced Heart Valve Centers, particularly for patients in whom surgery is unsuitable [60].

### 6.2. Indication—Clinical Circumstances in Which TAVI May Be Considered

- Severe, symptomatic chronic AR in patients at prohibitive or very high surgical risk (STS/EuroSCORE high, frailty, major comorbidities) where SAVR is not acceptable to the Heart Team and conservative therapy is inadequate [60].
- Predominant AR with unsuitable anatomy or comorbidities for surgery but with anatomies amenable to dedicated TAVI devices (appropriate annular dimensions, non-massively dilated root, adequate distance to coronaries) [61].
- Selected complex scenarios (prior cardiac surgery, prior CABG, prior mitral prosthesis, porcelain aorta) where a transcatheter solution may reduce procedural risk, provided careful imaging confirms feasibility [62].

An illustration example: a frail, multimorbid patient with severe AR, LV dilatation and NYHA III–IV, STS > 10%, and non-massive root dilatation, discussed by a Heart Team and allocated to TAVI with a dedicated device rather than redo sternotomy SAVR [60].

### 6.3. Contraindications—Features That Warrant Caution or Favor Consideration of Surgery

- Younger, low–intermediate surgical risk patients with isolated AR, who remain best served by surgical valve replacement given durability and evidence base [61].
- Massive aortic root/ascending aorta dilatation, severe leaflet prolapse or very eccentric jets leading to poor sealing and high risk of valve migration or significant paravalvular leak with current transcatheter systems [63].
- Very large, non-calcified annuli outside the sizing range of available transcatheter valves, or proximity of coronaries that precludes safe positioning and anchoring [60].
- Active endocarditis, thrombus in the LV or aortic root, or inability to obtain stable vascular access/adequate imaging guidance (TOE/CT) as for standard TAVI indications [64].

Overall, pure native AR without calcification is still considered a relative contraindication for conventional AS-designed TAVI systems; Heart Team selection and detailed imaging are mandatory [60].

### 6.4. Dedicated Devices—Dedicated Device Considerations and Limitations

- JenaValve (transapical and transfemoral) is the only system with extended CE-mark for pure native AR; it uses locator clips that grasp the native cusps, allowing anchoring independent of annular calcification and showing good acute success with low need for second valves and acceptable paravalvular leak [60].
- J-Valve (mainly used in China) employs U-shaped graspers to capture leaflets, providing self-positioning and stable fixation in non-calcified AR; observational data suggest high device success and favorable early hemodynamics [61].
- Other self-expandable platforms originally designed for AS (CoreValve/Evolut, ACURATE, Lotus, etc.) have been used off-label with oversizing and specific deployment strategies, but carry higher risks of malposition, embolization, and residual AR than dedicated AR devices. PubMed [61].

In systematic reviews, dedicated AR devices achieve higher technical success and lower rates of significant residual regurgitation or the need for a second valve compared with adapted AS valves, but long-term durability data remain limited [63]. Despite encouraging results with dedicated devices and contemporary registries, all current evidence for TAVI in native AR is nonrandomized and concentrated in a limited number of experienced centers, with no direct comparison versus surgery. TAVI for pure AR should therefore remain a niche, high-complexity therapy restricted to selected patients at prohibitive or very high surgical risk, treated at centers with specific expertise in AR-dedicated devices, and extrapolation to younger or intermediate-risk cohorts should be avoided until randomized data and long-term durability results are available.

## 7. TAVI in the Small Aortic Annulus

The central question in the small annulus—how best to maximize valve hemodynamics and avoid PPM—has been addressed by the most robust randomized evidence available in any of the three anatomies. The SMART trial randomized 716 patients with severe AS and an annular area ≤430 mm^2^ to a self-expanding supra-annular valve or a balloon-expandable valve. At 12 months, the self-expanding valve was non-inferior for the clinical composite of death, disabling stroke, or heart failure hospitalization and superior for the composite of bioprosthetic valve dysfunction; moderate or severe PPM at 30 days occurred in 11.2% of self-expanding versus 35.3% of balloon-expandable recipients [65]. Two-year data confirmed comparable clinical outcomes alongside sustained superiority of the self-expanding valve in valve performance, with significantly lower rates of prosthetic valve thrombosis and hemodynamic or structural valve dysfunction; five-year follow-up will determine whether these hemodynamic advantages translate into durability and clinical benefit [66]. Two-year SMART results were available as conference-reported data at the time of the literature search and should be interpreted as preliminary pending full peer-reviewed publication. These randomized data provide strong hemodynamic evidence favoring supra-annular self-expanding valves in small annuli, particularly regarding PPM and gradients. However, they do not yet demonstrate superiority in hard clinical outcomes or long-term durability, nor do they fully address lifetime scenarios such as redo-TAVI and valve-in-valve in very small prostheses. Recommendations to favor supra-annular self-expanding platforms in small annulus anatomy should therefore be framed primarily in terms of hemodynamic surrogates, with the understanding that lifetime management strategies will need to be refined as longer-term clinical data emerge.

In the randomized SMART trial, a self-expanding supra-annular valve was non-inferior to a balloon-expandable valve for the 12-month composite of death, disabling stroke, or heart failure rehospitalization (9.4% versus 10.6%), while demonstrating lower mean gradients, larger effective orifice area, and less bioprosthetic valve dysfunction and prosthesis–patient mismatch. These findings support a hemodynamic advantage of the studied supra-annular self-expanding strategy in patients with small annuli, but they do not establish superiority for long-term survival, stroke prevention, durability, or lifetime freedom from reintervention. Device selection should therefore be individualized according to annular and LVOT dimensions, coronary height and sinus/STJ anatomy, predicted coronary access and redo-TAVI feasibility, conduction system risk, vascular access, calcification pattern, risk of paravalvular regurgitation, and local operator experience with specific platforms. The 2-year SMART update reported sustained hemodynamic differences with similar clinical outcomes, but these results were conference reported and should be regarded as preliminary until available in a full peer-reviewed publication. Thus, a supra-annular self-expanding valve may be particularly attractive when avoidance of severe PPM or high residual gradients is the dominant objective, whereas an intra-annular or balloon-expandable platform may be more appropriate when coronary re-access, conduction risk, vascular considerations, or other patient-specific anatomical factors predominate.

These randomized findings are consistent with a large body of observational and meta-analytic evidence, although this literature reports predominantly hemodynamic surrogates rather than hard clinical endpoints. Pooled analyses spanning thousands of patients have shown that, compared with self-expanding valves, balloon-expandable valves are associated with a smaller indexed effective orifice area, higher transvalvular gradients, and a roughly two-fold higher risk of PPM and severe PPM in small annuli [67,68]; registry data from the STS/ACC TVT Registry established that PPM after supra-annular TAVI carries prognostic weight [69], and single-center series have reproduced the hemodynamic advantage of self-expanding (often supra-annular) designs, albeit with somewhat higher paravalvular leak rates [70,71]. Meta-analyses and matched studies further suggest that supra-annular rather than intra-annular leaflet positioning drives most of the benefit [72,73]. Superior gradients do not automatically translate into superior long-term survival, which remains the central interpretive caveat, and the prognostic impact of PPM has been more consistent in surgical than in transcatheter cohorts [74].

Surgery remains a relevant comparator, particularly for younger, lower-risk patients for whom lifetime management is central. Rapid-deployment and sutureless bioprostheses can provide favorable hemodynamics in small annuli, and surgical aortic annular enlargement—from the classic Nicks and Manouguian techniques to the more radical Y-incision (roof) enlargement—allows implantation of a larger prosthesis and may mitigate PPM and facilitate future valve-in-valve procedures, at the cost of added surgical complexity [75]. Unanswered questions include the ideal device for the extra-small annulus, the long-term durability and reintervention burden of a small index prosthesis, the cumulative lifetime hemodynamic cost of any residual gradient, and—critically—the feasibility of future valve-in-valve TAVI when the index valve is already small. The landmark studies underpinning the evidence base for TAVI in bicuspid aortic stenosis, pure native aortic regurgitation, and small aortic annulus are summarized in Table 5.

In the small annulus, surgery offers two major lifetime advantages that are only partially replicated by transcatheter strategies: (i) annular/root enlargement and (ii) use of rapid-deployment or sutureless valves. Classical enlargement procedures (Nicks posterior enlargement, Manouguian anterior enlargement, and modern Y-incision/rectangular patch techniques) can increase annular size sufficiently to implant a larger surgical prosthesis, thereby reducing the risk of severe PPM and creating a more favorable landing zone for future valve-in-valve procedures. Contemporary series report acceptable operative risk in experienced centers, but at the cost of longer cross-clamp times and the need for advanced surgical expertise; data in elderly, frail patients are more limited and heterogeneous.

Rapid-deployment and sutureless valves provide enlarged effective orifice area and shorter cross-clamp times compared with conventional stented bioprostheses, and they are attractive in small annuli from a hemodynamic standpoint. However, they introduce specific lifetime constraints: valve-in-valve procedures into these prostheses may have higher residual gradients, and the rigid frame can complicate future coronary access and valve explantation.

By contrast, a supra-annular self-expanding TAVI prosthesis can achieve excellent hemodynamics and low rates of severe PPM in small annuli, often with lower early risk in elderly patients, but does not enlarge the annulus and may restrict future coronary access depending on frame height and commissural orientation. From a lifetime planning perspective, surgery with annular enlargement is generally preferred in younger, low-risk patients with very small annuli and long life expectancy, whereas TAVI with a supra-annular device is reasonable in older or high-risk patients in whom annular enlargement would confer limited long-term benefit relative to its procedural cost.

### 7.1. Take-Home Message

These randomized data provide strong hemodynamic evidence favoring supra-annular self-expanding valves in small annuli, particularly regarding and gradients. However, they do not yet demonstrate superiority in hard clinical outcomes or long-term durability, nor do they fully address lifetime scenarios such as redo-TAVI and valve-in-valve in very small prostheses. Recommendations to favor supra-annular self-expanding platforms in small annulus anatomy should therefore be framed primarily in terms of hemodynamic surrogates, with the understanding that lifetime management strategies will need to be refined as longer-term clinical data emerge. From a device strategy perspective, supra-annular self-expanding valves may offer lower gradients and a lower risk of moderate or severe PPM in selected small-annulus anatomies. However, device selection should remain individualized, incorporating coronary and root anatomy, predicted coronary re-access, pacemaker risk, vascular access, calcification distribution, risk of paravalvular regurgitation, and anticipated lifetime reintervention strategy. In selected patients with a small aortic annulus, the most robust comparative evidence supports a hemodynamic advantage of supra-annular self-expanding TAVI over balloon-expandable TAVI, particularly with respect to indexed effective orifice area, residual gradients, and prosthesis–patient mismatch. This advantage should not be interpreted as definitive superiority for hard clinical outcomes, long-term durability, coronary re-access, or lifetime treatment strategy; the choice between TAVI and surgery remains individualized.

### 7.2. Indication—When a Small Annulus Favors TAVI—Table 6

(1) Elderly or high-surgical-risk patients with small annulus and anticipated severe PPM with surgical bioprosthesis, in whom transcatheter supra-annular valves can provide larger effective orifice areas and lower gradients. (2) Patients with small annuli and comorbidities (frailty, COPD, CKD, prior cardiac surgery) where TAVI is already preferred, and annular dimensions further strengthen the case for a transcatheter rather than surgical approach. (3) Borderline annular sizes where surgery would necessitate complex root enlargement procedures that carry added operative risk, but transfemoral TAVI can achieve acceptable hemodynamics without annular enlargement.

**Table 6:** Proposed device selection algorithm in small anulus.

Dominant Consideration	Device Selection Implication
High predicted PPM or elevated residual-gradient risk	Consider a supra-annular self-expanding platform, provided coronary, access, and conduction considerations are acceptable.
Low coronary height, narrow sinuses, adverse STJ anatomy, or likely future coronary intervention	Prioritize valve frame design, commissural orientation, implant depth, and predicted coronary re-access rather than hemodynamics alone.
High conduction system risk or anatomy favoring controlled deployment	Consider device-specific implantation characteristics and pacemaker risk trade-offs; avoid a uniform platform recommendation.
Unfavorable iliofemoral access	Select the treatment modality and access route according to vascular anatomy; small annulus alone should not determine device choice.
Younger patient with a long anticipated treatment horizon	Integrate the feasibility of future PCI, redo-TAVI, surgical explant, and possible annular enlargement into the initial Heart Team decision.

### 7.3. Contraindications and Red Flags

(1) Extremely small annuli outside the validated sizing range of any available THV or with unfavorable LVOT geometry (e.g., very narrow, septal bulge) where any reasonable TAVI strategy would risk annular rupture or severe PPM. (2) Need for reliable future coronary access in younger or intermediate-risk patients, particularly with low coronary take-off and short valve–coronary distance, where high-frame TAVI devices may jeopardize re-access; surgery with tailored prosthesis remains preferred. (3) Multilevel left-sided obstruction (subvalvular, valvular) or complex root pathology where treating only the valve with TAVI will not resolve the hemodynamic problem.

### 7.4. Device and Procedural Strategy—Key Points

(1) Favor supra-annular self-expanding platforms when possible, as they typically give larger indexed effective orifice areas and lower PPM risk than balloon-expandable valves in a small annulus. (2) Avoid excessive oversizing or aggressive post-dilation; in a truly small, rigid annulus, the priority is preventing rupture, accepting a modest gradient if necessary rather than chasing “perfect” hemodynamics. (3) Use high-quality CT to integrate annular area, perimeter, LVOT dimensions, sinus and STJ size, and coronary height; Heart Team discussion should explicitly weigh PPM risk versus rupture and future coronary access.

## 8. Cross-Cutting Issues and Lifetime Management

The anatomies reviewed here converge on a shared imperative: because the patients are younger, the index procedure must be planned as the first of a possible sequence of interventions. Table 6 maps how each anatomy influences the principal lifetime management domains. Lifetime management of aortic valve disease in bicuspid anatomy, small annulus and predominant regurgitation requires an explicitly longitudinal, “lifetime procedures” mindset rather than a single-episode decision. Across these phenotypes, baseline CT and echo must be interpreted not only for immediate TAVI feasibility (annulus, root, coronaries) but also for how future coronary access, valve-in-valve options, and potential aortic surgery will be influenced [77]. Bicuspid valves and small annuli share a high risk of PPM and challenging coronary re-access, so device choice, frame height and commissural alignment should be tailored with expected life expectancy and re-intervention scenarios in mind. In pure or predominantly AR and in less calcified bicuspid valves, the limitations of current AS-designed TAVI platforms (anchoring, migration, PVL) mean the threshold for surgery remains lower, and dedicated AR devices or careful surgical planning must be integrated into the lifetime strategy [78]. Small annulus patients, especially younger or intermediate risk, often benefit from surgical strategies that optimize future options (annular/root enlargement, choice of prosthesis) rather than “locking in” a difficult TAVI scenario. For all three phenotypes, serial imaging follow-up (echo, CT when appropriate) is not only to assess valve function, but also to track root/ascending aorta dimensions, coronary access corridors, and suitability for valve-in-valve or alternative transcatheter therapies [79]. Antithrombotic and endocarditis prevention strategies should be individualized, acknowledging that multiple interventions and prostheses over a lifetime increase cumulative risk. Shared decision-making must explicitly include discussion of durability, likely need and mode of re-intervention (surgery vs. TAVI-in-TAVI vs. alternative devices), and how each current choice constrains future ones. Finally, these complex anatomies should be managed in centers with Heart Teams experienced in both advanced surgery and contemporary TAVI platforms, so initial and subsequent interventions remain coherent within a deliberately planned lifelong care pathway [80]. Age and projected life expectancy explicitly modify the framework. In younger patients or those expected to outlive an index bioprosthesis, the Heart Team should give greater weight to durability uncertainty, future coronary access, redo-TAVI feasibility, surgical explant complexity, and whether surgery could provide a more future-proof initial strategy through aortic repair or annular/root enlargement. In older patients or those with limited life expectancy, avoidance of surgical trauma and optimization of immediate hemodynamic and symptomatic benefit may carry greater weight, provided that anatomical suitability is adequate.

### 8.1. Coronary Access and Obstruction

Bulky bicuspid leaflets, narrow sinuses, and the small annulus all raise the risk of acute coronary obstruction and of difficult selective coronary cannulation later in life [81]. The problem is compounded when a tall THV frame displaces bulky leaflets toward the ostia or creates a neoskirt across the sinus. Coronary access and obstruction in the setting of bicuspid anatomy, small annulus, AR and TAVI must be considered as a lifetime issue, not just a peri-procedural complication. Pre-procedural CT should systematically assess coronary height, sinus and sinotubular junction dimensions, valve-to-coronary and valve-to-aorta distances and predict both immediate obstruction risk and future feasibility of coronary re-access or redo-TAVI. Small annulus and small sinotubular junction, especially with supra-annular THV designs, are strongly associated with impaired future coronary access and increased risk of coronary obstruction in valve-in-valve or TAVI-in-TAVI scenarios. In bicuspid or AR phenotypes, eccentric leaflet calcification, bulky leaflets, and low coronary take-off further increase obstruction risk, demanding tailored device choice, implantation height, and consideration of commissural alignment to preserve access corridors. Coronary obstruction remains rare (~1% in large series) but carries very high early mortality, so identification of high-risk anatomy and planned preventive strategies (coronary protection, with guidewires or undeployed stents, Chimney stenting [82,83], BASILICA [84,85]) are essential. For patients likely to require future PCI or redo-TAVI, Heart Teams should favor THV platforms and implantation strategies that optimize valve-to-coronary distance and commissural alignment, even at the expense of some hemodynamic “perfection.” Redo-TAVI CT data show that more than half of patients may have impaired coronary access and 10–20% at increased obstruction risk, underscoring the need to “plan ahead” at the index procedure. When lifetime PCI needs are anticipated (younger, CAD-burdened patients), SAVR with an appropriately sized and positioned prosthesis may remain preferable to TAVI to avoid creating an inaccessible coronary environment. Finally, complex cases (bicuspid, small annulus, AR) with high predicted obstruction risk should be concentrated in centers experienced with CT-guided planning and advanced protective techniques, ensuring coronary safety is integrated into the overall lifetime management strategy [82]. The suggestion that Heart Teams may accept modest hemodynamic compromise to preserve future coronary access is conceptually important but must be operationalized. In younger patients with high lifetime coronary disease risk or existing coronary grafts, prioritizing access may justify choosing a slightly smaller frame or an intra-annular device with marginally higher gradients but wider open cells and more predictable coronary cannulation, or accepting mild PPM in exchange for a lower skirt and shorter frame. This trade-off is particularly relevant in bicuspid anatomy with asymmetric sinuses and in small annuli, where supra-annular devices may provide superior gradients but at the cost of taller frames and more constrained sinus space.

### 8.2. Commissural Alignment

Commissural alignment during TAVI has emerged as a key determinant of lifetime valve and coronary management, particularly in patients with small annuli, bicuspid stenosis, and pure aortic regurgitation [86]. In small-annulus anatomy, achieving commissural alignment is crucial to optimize the effective orifice area and reduce patient–prosthesis mismatch, thereby mitigating heart failure risk and early structural valve degeneration [86]. In bicuspid aortic valve stenosis, where asymmetric calcification and elliptical annuli predispose to prosthesis underexpansion and eccentric deployment, neo-commissural alignment may improve device–root interaction and facilitate future coronary access [24]. Pre-procedural multidetector CT with assessment of native commissural orientation, coronary ostia height, sinotubular junction geometry and intercommissural distance forms the basis for patient-specific planning of commissural alignment in these complex anatomies. Modified delivery system insertion and rotation techniques, guided by CT–fluoroscopy co-registration and standardized markers (for example, ALIGN-TAVR criteria and hat marker orientation for self-expanding valves), have been shown to improve commissural alignment and reduce severe coronary overlap [87]. In balloon-expandable platforms, tailored fluoroscopic views and crimping orientation allow relatively precise commissural and coronary alignment, which is particularly advantageous in small annulus and bicuspid valves where coronary access may otherwise be at high risk [86]. Emerging data suggest that commissural misalignment independently predicts hypo-attenuated leaflet thickening and subclinical leaflet thrombosis after TAVI, underscoring the hemodynamic and durability implications of alignment beyond coronary access alone [88]. In pure native aortic regurgitation, where anchoring is more challenging and oversizing is often required, commissural alignment may help distribute radial forces more symmetrically, potentially improving sealing and reducing paravalvular regurgitation [89]. Across small annulus, bicuspid stenosis, and aortic regurgitation cohorts, an individualized device selection and implantation strategy that prioritizes commissural alignment is recommended to preserve coronary reaccess, enable safe redo-TAVI, and optimize long-term valve performance. Future refinement of transcatheter heart valve and delivery system designs is expected to make neo-commissural alignment more reproducible in complex anatomy, thereby integrating alignment as a standard procedural goal in contemporary TAVI practice [86].

### 8.3. Redo-TAVI and Surgical Explant

Redo-TAVI has emerged as the preferred reintervention strategy for failed transcatheter valves because it offers lower peri-procedural mortality and morbidity than surgical explant, even in anatomically complex cohorts [90]. In patients with a small aortic annulus, supra-annular self-expanding platforms are generally favored for redo-TAVI, as they improve the effective orifice area and reduce the risk of patient–prosthesis mismatch compared with intra-annular designs [91]. However, small annulus anatomy also increases the risk of coronary obstruction and impaired coronary access at redo-TAVI, making meticulous pre-procedural CT planning and commissural alignment crucial for lifetime management [92]. Bicuspid aortic stenosis poses additional challenges for redo-TAVI due to asymmetric calcification, elliptical annular geometry, and frequent aortopathy, all of which can affect anchoring, device expansion, and long-term durability [93]. Contemporary data suggest that redo-TAVI in bicuspid valves is feasible, but careful device selection and implant depth are essential to preserve coronary access and minimize the risk of root injury or severe patient–prosthesis mismatch. For predominant native or prosthetic aortic regurgitation, redo-TAVI is technically more demanding because of the lack of calcification and high stroke volume, requiring devices with strong radial force, anchoring features, and often rapid pacing or cerebral protection to reduce embolic risk [94].

Surgical explant of a transcatheter valve and conversion to surgical AVR remain the definitive option when redo-TAVI is contraindicated or unsafe, such as in severe prosthesis under-expansion, infective endocarditis, extensive pannus, or prohibitive coronary obstruction [95]. Nevertheless, contemporary series consistently report very high operative risk for THV explant, with notable early mortality and frequent need for complex root procedures, underscoring the importance of careful lifetime planning at the index TAVI. In patients with a small annulus, bicuspid anatomy, or pure aortic regurgitation, the Heart Team should integrate annular dimensions, valve type and position, coronary anatomy, and patient age to choose between redo-TAVI and surgical explant within a structured lifetime management algorithm. Future research should refine device-specific strategies for redo-TAVI in these high-risk anatomies and better define when early surgical explant might offer superior hemodynamics and long-term outcomes despite its short-term risk profile [90].

### 8.4. Durability and Aortopathy

Long-term data suggest that transcatheter bioprostheses show acceptable structural durability for 5–10 years, but durability becomes a major concern in younger patients and those with longer life expectancy [96]. In small-annulus patients, durability is closely linked to prosthesis hemodynamics, as high residual gradients and severe patient–prosthesis mismatch accelerate structural valve degeneration and increase the risk of bioprosthetic valve failure [97]. Recent series with balloon-expandable valves report comparable 7-year durability in small versus large annuli, indicating that annular size per se may be less important than valve sizing, expansion, and residual gradient [1]. In bicuspid aortic stenosis, early and mid-term studies show that TAVI durability is broadly similar to tricuspid anatomy, but asymmetric loading, under-expansion, and elliptical geometry may predispose to earlier degeneration in some patients. Moreover, bicuspid aortic valve disease is frequently associated with aortopathy involving dilation of the root and ascending aorta, which can progress independently of valve intervention and influence long-term outcomes and reintervention strategies [98]. Current ACC/AHA recommendations emphasize systematic imaging follow-up of bicuspid-associated aortopathy, with intervention thresholds based on aortic diameter, growth rate, family history, and concomitant valve surgery [99].

In patients with predominant aortic regurgitation, both native and prosthetic, durability is strongly influenced by secure anchoring and full expansion of the transcatheter valve, as chronic volume overload and lack of calcification can promote malpositioning and leaflet stress [96]. Persistent or recurrent aortopathy, including progressive ascending aortic dilatation or aneurysm formation, may ultimately require surgical correction even when valve durability remains acceptable, particularly in bicuspid phenotypes [78]. Therefore, lifetime management in small-annulus bicuspid stenosis and aortic regurgitation must integrate valve durability data with individualized aortic imaging surveillance and clear thresholds for prophylactic aortic surgery. Future long-term registries and randomized studies are needed to better define durability patterns of different TAVI platforms in these complex anatomies and to refine combined valve–aorta strategies across the patient lifespan [96]. The proposed framework integrates three interdependent domains. The first domain is the anatomical substrate, defined by ECG-gated CT and echocardiography, including valve morphology, annular and supra-annular dimensions, calcium distribution, LVOT anatomy, aortic root and ascending aortic dimensions, coronary relationships, and vascular access. The second domain is procedural and device strategy, including feasibility of anchoring, device platform, sizing, implantation depth, commissural alignment, coronary protection strategy, and bailout options. The third domain is the lifetime management horizon, incorporating age, estimated life expectancy, surgical risk, frailty, valve durability, future coronary access, redo-TAVI feasibility, surgical explantation, valve-in-valve options, and surveillance or repair of associated aortopathy. The final treatment recommendation should be determined by the intersection of all three domains in a multidisciplinary Heart Team discussion. AR, aortic regurgitation; BAV, bicuspid aortic valve; LVOT, left ventricular outflow tract; PPM, prosthesis–patient mismatch; SAVR, surgical aortic valve replacement; TAVI, transcatheter aortic valve implantation. This is illustrated in Figure 1.

## 9. Overlap Phenotypes (When Complex Anatomies Coexist)

Overlap phenotypes require an integrated assessment because the dominant anatomical diagnosis does not always identify the dominant procedural hazard. In each case, the Heart Team should first identify the principal mechanical limitation—anchoring/sealing failure, under-expansion or PPM, coronary obstruction, or concomitant aortopathy—and then determine whether a transcatheter strategy would leave an important pathology untreated. Three illustrative combinations merit particular attention. In bicuspid AS with a small annulus, the potential hemodynamic benefit of a supra-annular device must be balanced against asymmetric expansion, coronary access, and any aortic surgical target. In AR with root or ascending aortic dilatation, surgery may be more comprehensive when both the valve lesion and aortopathy require treatment. In AR with borderline annular dimensions, the dynamic anchoring environment and the margin within device sizing instructions should be assessed carefully; if a secure transcatheter result is uncertain and surgical risk is acceptable, surgery should be actively discussed. These examples are not decision rules. They illustrate why overlap anatomy should be assessed in experienced Heart Valve Centers using multimodality imaging, guideline-based surgical thresholds, and individualized shared decision-making. “Figure operationalizes this approach for overlapping phenotypes. When more than one complex anatomical feature is present, treatment selection should begin with identification of the dominant mechanical constraint—anchoring/sealing failure, limited expansion and severe PPM risk, coronary obstruction or future coronary access limitation, or untreated aortopathy—rather than with assignment to a single anatomical category. The final strategy should then integrate surgical candidacy, age and expected lifetime treatment horizon, device-specific feasibility, and the impact of the index intervention on future coronary and valvular procedures—Figure 2.

## 10. Limitations of the Current Evidence and of This Review

The evidence synthesized here has important constraints, and so does the review itself. First, the literature is dominated by observational and single-arm data, especially for pure AR, where no randomized comparison against surgery or between device classes exists; even the registries that appear reassuring in bicuspid AS are subject to selection of anatomically favorable candidates, treatment at expert centers, and residual confounding that propensity methods cannot fully remove. Second, anatomy definitions, calcium thresholds, and endpoint definitions have varied across studies and device generations, and harmonized VARC-3 reporting has been applied only recently [18]; pooled analyses therefore combine heterogeneous populations and valve iterations. Third, follow-up rarely extends beyond five years, which is inadequate for a durability question in patients who may live for two decades, and several of the most influential recent datasets (including the SMART two-year and NOTION-2 three-year analyses) are still maturing. Fourth, many favorable outcomes derive from high-volume centers and may not generalize to all programs. Historical comparisons should therefore be interpreted cautiously, as ongoing device iteration and operator learning curve effects can shift procedural success, complication profiles, and hemodynamic outcomes over time.

This review is itself narrative rather than systematic: it did not apply a formal risk-of-bias instrument or quantitative pooling, its search—though structured and multi-database—was restricted to English-language sources and may have missed grey literature, and evidence prioritization, while explicit, involved author judgement. The proposed anatomy-driven framework is a synthesis intended to support decision-making, not a validated algorithm, and should be tested prospectively before being treated as a standard of care.

## 11. An Anatomy-Specific Research Agenda

For bicuspid AS, the priorities are a standardized, prospectively validated CT-based sizing algorithm; adequately powered randomized comparisons of THV types and of TAVI versus surgery, specifically in bicuspid anatomy, with central adjudication of morphology to exclude unicuspid valves; and long-term durability and aortopathy progression data in patients treated in their sixties. A pragmatic near-term design would be an anatomy-stratified RCT with core-lab imaging and pre-specified bicuspid subgroups powered for clinical—not merely hemodynamic—endpoints.

For pure AR, the field needs randomized comparisons of dedicated devices against surgery and against non-dedicated transcatheter approaches; a consensus definition of the anchoring zone and of sizing methodology in non-calcified anatomy; and durability and late-failure data as dedicated devices diffuse into practice following regulatory approval. Registry-embedded randomization at experienced centers may be the only feasible route given device access and patient numbers.

For the small annulus, the outstanding questions are randomized comparisons of device types and of transcatheter versus surgical strategies (including annular enlargement) with hemodynamic and clinical endpoints; the optimal approach to the extra-small annulus; the quantification of the lifetime hemodynamic burden of residual PPM; and the feasibility and outcomes of future valve-in-valve procedures when the index prosthesis is small. Cutting across all three, the discipline requires long-term durability data in complex anatomy, harmonized VARC-3 outcome reporting, and collaborative registries and trials powered for anatomy-specific subgroups rather than the pooled cohorts that have characterized the field to date.

## 12. Practical Implications for Heart Teams

Translating this evidence into practice requires the Heart Team to integrate three axes simultaneously: the anatomical substrate, the patient’s age and surgical risk, and the mechanical characteristics of the available devices. After anatomical characterization, the framework should classify patients according to the anticipated lifetime treatment horizon: shorter, intermediate, or long. This classification is informed by age, estimated life expectancy, frailty, comorbidity, and expected future coronary and valvular intervention needs, rather than chronological age alone.

A pragmatic, ordered pathway that a Heart Team can follow during case review is:

1. Define the anatomy on ECG-gated CT using the checklist in Table 2, and classify the dominant lesion as bicuspid AS, pure AR, or small-annulus AS (recognizing that these can coexist).
2. Ask whether the substrate favors a particular device class: a supra-annular self-expanding valve for the small annulus and for many asymmetric bicuspid valves; a dedicated leaflet-clasping device for pure AR.
3. Assess surgical candidacy and life expectancy: for younger, operable patients—especially bicuspid anatomy with a calcified raphe or aortopathy, pure AR with root dilation, or a small annulus amenable to enlargement—weigh surgery explicitly.
4. Overlay lifetime management constraints: coronary access, commissural alignment, and redo/explant feasibility, choosing index valve type, size, depth, and orientation with the next procedure in mind.
5. Concentrate complex-anatomy cases in experienced Heart Valve Centers, consistent with the 2025 guidelines’ emphasis on the volume–outcome relationship and structured, CT-driven protocols.

Proposed algorithm can be funded in Supplementary Materials S3.

## 13. Conclusions

In patients with complex aortic valve anatomy—including bicuspid aortic stenosis, pure native aortic regurgitation, and a small aortic annulus—TAVI is technically feasible and can provide acceptable early and mid-term outcomes when performed in carefully selected patients at experienced Heart Valve Centers. However, the evidence supporting its use is substantially less mature than that for tricuspid calcific aortic stenosis and differs importantly among these anatomical substrates.

In bicuspid aortic stenosis, treatment selection should be driven by CT-defined valve morphology, raphe and leaflet calcification, annular and supra-annular geometry, coronary anatomy, and associated aortopathy; surgery remains particularly compelling when unfavorable morphology or a concomitant aortic indication is present. In pure native aortic regurgitation, dedicated leaflet-anchoring devices have improved procedural feasibility, but available evidence remains predominantly single-arm and observational; therefore, surgery remains the standard approach for operable patients, while TAVI should be reserved for selected patients unsuitable for surgery with appropriate anatomy. In small annuli, supra-annular self-expanding TAVI provides a consistent hemodynamic advantage and reduces prosthesis–patient mismatch compared with balloon-expandable TAVI, although definitive evidence of superiority for long-term clinical outcomes, durability, coronary re-access, and lifetime freedom from reintervention is lacking.

The principal contribution of this review is an anatomy-driven framework that integrates CT-based phenotyping with device selection, implantation strategy, surgical alternatives, and lifetime management considerations, including coronary access, redo-TAVI feasibility, surgical explantation, and surveillance of associated aortopathy. The initial intervention should be planned not as an isolated procedure, but as the first step in a potential lifetime sequence of valve and coronary interventions. Future progress will require standardized CT-based sizing and anchoring criteria, anatomy-specific randomized trials, harmonized VARC-3 reporting, and long-term data on durability, aortic disease progression, coronary access, and reintervention strategies.

## Figures and Tables

**Figure 1:**
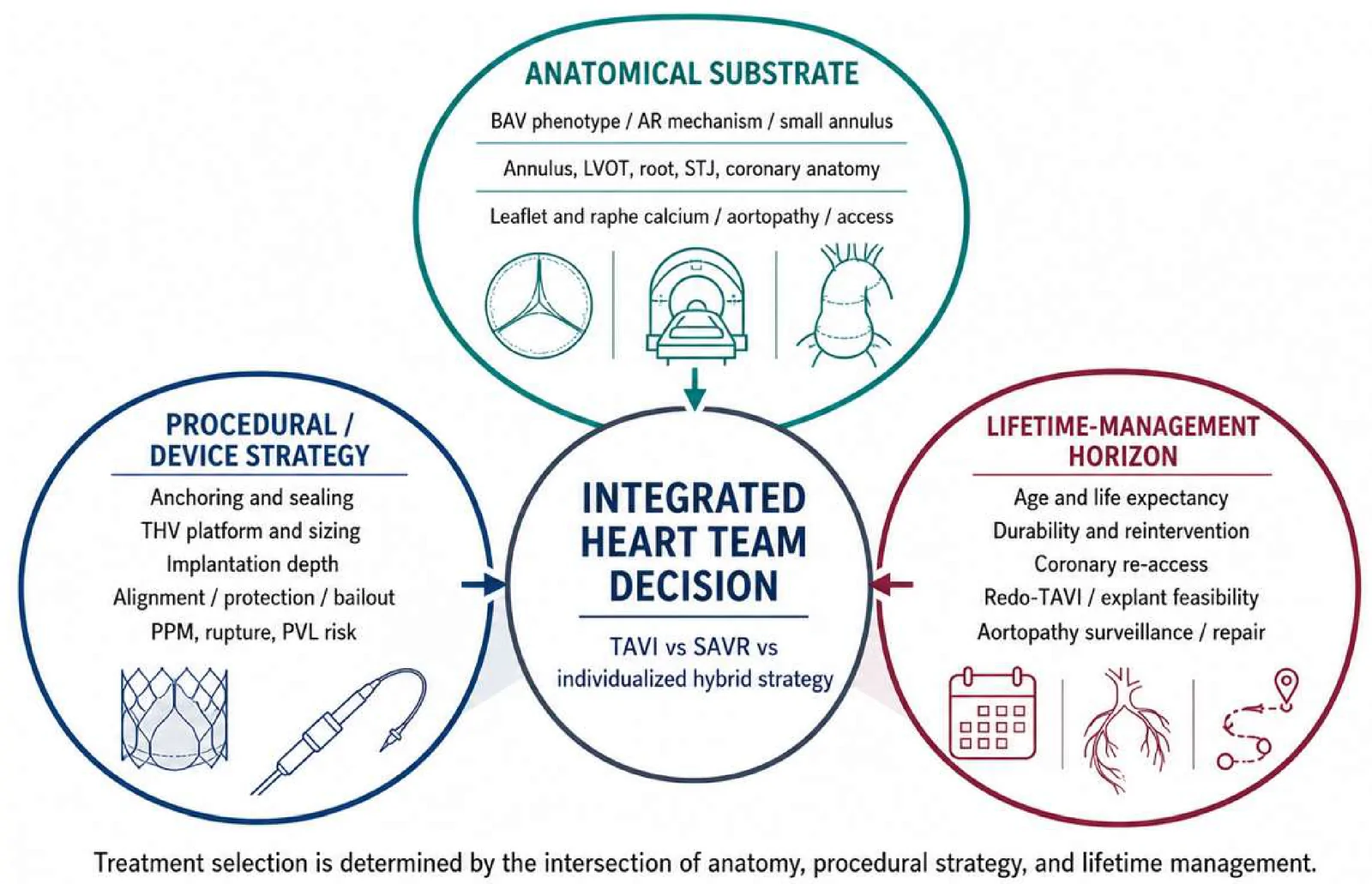
Three-dimensional framework for treatment selection and lifetime management in complex aortic valve anatomy.

**Figure 2:**
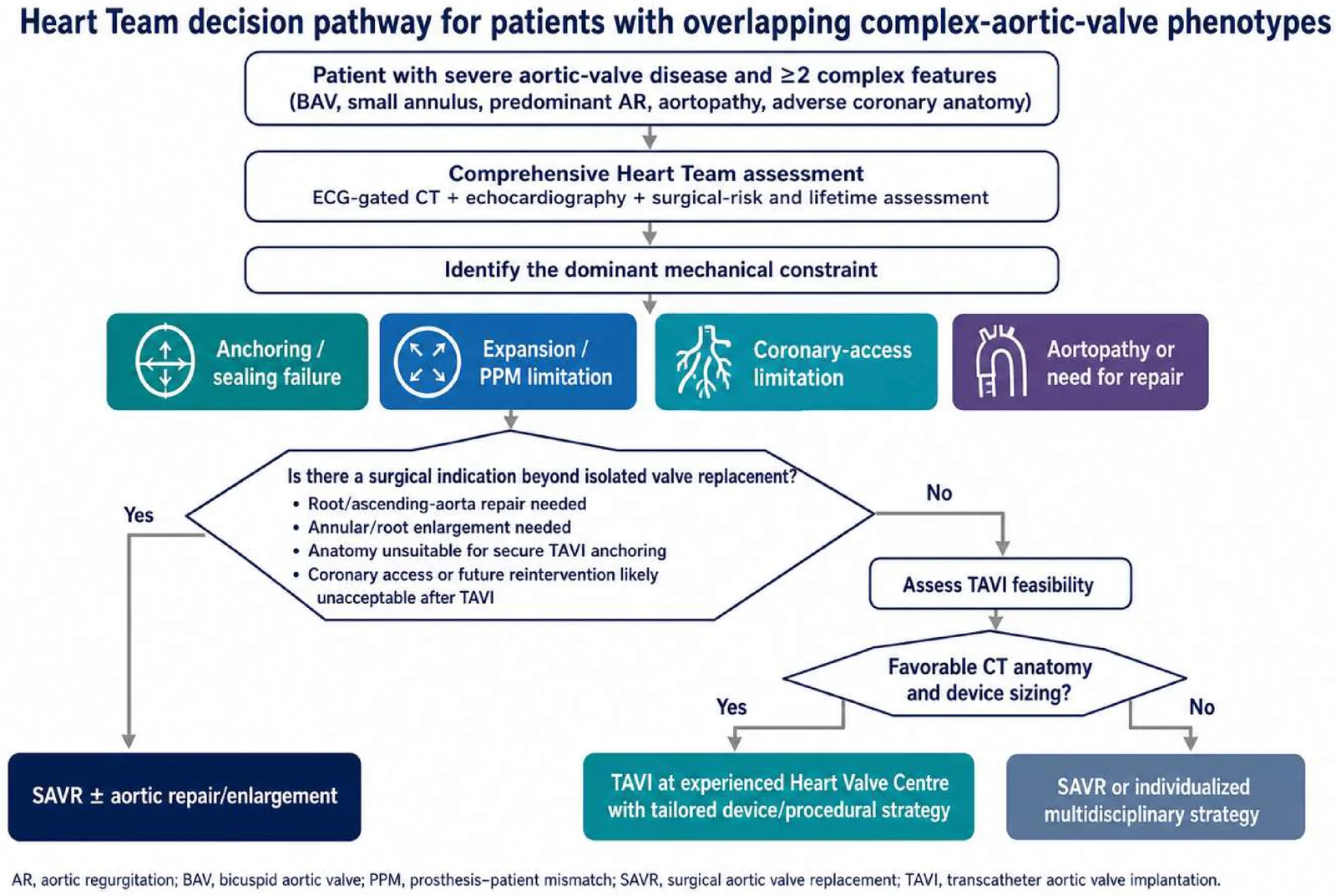
An anatomy-driven heart team pathway for overlapping complex aortic valve phenotypes.

**Table 1:** Anatomy-driven framework for transcatheter aortic valve implantation in bicuspid aortic stenosis, pure native aortic regurgitation, and the small aortic annulus.

Anatomy	Core Anatomical/Mechanical Challenge	Imaging Priority	Treatment/Device Implications	Principal Procedural and Lifetime Hazards	Key Evidence Gaps
Bicuspid AS	Elliptical annulus and supra-annular constraint; asymmetric leaflet/raphe calcification; frequent concomitant aortopathy. A calcified raphe combined with excess leaflet calcification identifies a high-risk phenotype.	Define BAV morphology; compare annular and supra-annular dimensions; assess raphe and leaflet calcium distribution, LVOT calcium, coronary anatomy, and root/ascending-aortic dimensions.	Individualized THV selection and sizing; repositionable or supra-annular SEV may be useful in selected anatomies. Surgery is generally preferred in younger/operable patients with heavily calcified raphe, bulky asymmetric calcium, or an aortic indication.	Under-expansion, paravalvular leak, aortic root injury, conduction disturbance, coronary obstruction/re-access limitation, and untreated aortopathy.	Anatomy-specific randomized trials; validated CT-based sizing algorithms; long-term durability; optimal management and surveillance of associated aortopathy.
Pure native AR	Distinguish leaflet-predominant AR from functional AR caused by annular, root, or ascending aortic dilatation. Lack of calcific anchoring, a large/dynamic annulus, and persistent aortopathy may coexist.	Define cusp morphology and coaptation; assess annulus–LVOT–STJ anchoring zone, systolic annular dimensions, root/ascending aortic dimensions, coronary anatomy, and the need for concomitant aortic repair.	Consider a dedicated leaflet-anchoring THV only when there is a suitable anchoring substrate and surgery is unsuitable. Favor surgery when root/ascending aortic repair is indicated, annular dimensions exceed device range, or an adequate anchoring zone is absent.	Malposition, migration/embolization, residual AR, second-valve implantation, conduction disturbance, coronary obstruction, and failure to treat progressive aortopathy.	Randomized comparisons with surgery; dedicated versus non-dedicated device comparisons; validated mechanism-specific sizing/anchoring criteria; long-term durability and aortopathy outcomes.
Small annulus	Restricted effective orifice area and elevated risk of prosthesis–patient mismatch. An annular area <430 mm^2^ identifies the broader trial-defined small-annulus group; approximately <400 mm^2^ may identify a particularly small/high-risk subgroup.	Annular area, perimeter, and diameters; LVOT dimensions and calcium; body surface area and predicted indexed effective orifice area; coronary height, sinus/STJ dimensions, and future valve-in-valve feasibility.	Favor a supra-annular SEV when compatible with coronary anatomy and lifetime strategy to optimize effective orifice area and reduce PPM. Consider SAVR with annular/root enlargement in younger, low-risk patients with very small annuli or an unfavorable lifetime TAVI trajectory.	High residual gradient, moderate/severe prosthesis–patient mismatch, annular rupture with aggressive oversizing, coronary obstruction, impaired coronary re-access, and constrained future valve-in-valve options.	Management of extra-small annuli; TAVI versus SAVR with annular enlargement; long-term clinical effect of residual PPM; durability; future redo-TAVI and coronary access outcomes.

Abbreviations: AR, aortic regurgitation; AS, aortic stenosis; BAV, bicuspid aortic valve; CT, computed tomography; LVOT, left ventricular outflow tract; PPM, prosthesis–patient mismatch; SAVR, surgical aortic valve replacement; SEV, self-expanding valve; STJ, sinotubular junction; TAVI, transcatheter aortic valve implantation; THV, transcatheter heart valve.

**Table 2:** Suggested CT planning checklist for complex-anatomy TAVI. Items in bold are anatomy-specific priorities.

Domain	Measurement	Why It Matters/Anatomy Emphasis
Annulus	Area, perimeter, mean diameter, eccentricity	Sizing; PPM prediction (small annulus); oversizing (AR)
LVOT	Area, calcium, membranous septum length	Annular rupture and pacemaker risk; small-annulus PPM
Leaflets/raphe	Leaflet length, calcium bulk, raphe calcification	Bicuspid sizing and expansion; anchoring in AR
Coronary heights	Left and right ostial height	Coronary obstruction risk; future re-access
Sinus/STJ	Sinus width, STJ height and diameter	Anchoring zone (AR); sinus sequestration at redo
Aorta	Ascending aorta and root diameters	Bicuspid aortopathy; surgical-vs-TAVI decision
Access	Ilio-femoral caliber and calcification; arch	Vascular complication risk; alternative access
Fluoroscopy	Predicted deployment and cusp-overlap angles	Depth control and commissural alignment

Abbreviations: AR, aortic regurgitation; CT, computed tomography; LVOT, left ventricular outflow tract; PPM, prosthesis–patient mismatch; STJ, sinotubular junction.

**Table 3:** Bicuspid aortic valve phenotypes and procedural strategy.

Phenotype	Procedural Risk Signal	Evidence-Informed Considerations for Heart Team Discussion
Symmetric type 1 bicuspid valve with low calcium burden	More circular deployment environment, lower likelihood of major frame distortion or relevant paravalvular leak compared with heavily calcified or asymmetric phenotypes; contemporary reviews describe this morphology as among the more suitable bicuspid anatomies for transcatheter treatment, particularly when CT sizing is favorable [41]	Favorable for TAVI, especially with self-expanding or supra-annular platforms when anatomy is otherwise suitable; in low-surgical-risk patients, treatment choice should be individualized through structured Heart Team and shared decision-making [42,43].
Type 0 bicuspid valve or very elliptical annulus, with low calcium burden and no relevant aortopathy	Elliptical geometry increases the risk of sizing error, incomplete expansion, or sealing difficulty, even when calcium burden is limited; CT-based annular and supra-annular assessment is therefore [44]	TAVI is feasible in expert centers when calcium burden is low and the aorta is not a surgical target, but meticulous multimodality imaging, careful sizing, and procedural planning are mandatory [45]
Calcified raphe or bulky asymmetric leaflet/LVOT calcium	Registry data show higher risks of under-expansion-related device failure patterns, paravalvular regurgitation, aortic root injury, and worse clinical outcomes when calcified raphe and excess leaflet calcification are present [42]	In low-risk patients, surgery is generally preferred because severe asymmetric calcification and calcified raphe represent adverse anatomical features for TAVI [46]
Significant ascending aortic dilatation	Concomitant aortopathy changes the procedural objective beyond valve replacement alone; bicuspid aortic valve surgery literature supports addressing the ascending aorta when operative thresholds are met because valve-only therapy leaves the aortic pathology untreated [47].	Surgery with concomitant ascending aortic repair is indicated when clinically significant ascending aortic dilatation is present, regardless of whether the valve lesion might otherwise appear technically suitable for TAVI [45].
Calcified raphe plus excess leaflet calcification	High-risk CT phenotype associated with constrained/asymmetric THV expansion, impaired sealing, moderate/severe PVL, aortic root injury, and higher mortality. Risk may be amplified by marked annular eccentricity, LVOT calcium, or aggressive oversizing/post-dilatation [39]	Surgery generally preferred in younger or operable patients. If TAVI is required, use detailed CT-based sizing, avoid excessive oversizing, individualize pre-/post-dilatation, consider cerebral protection when embolic risk is high, and perform the procedure in an experienced Heart Valve Centre [2]

Abbreviations: CT, computed tomography; LVOT, left ventricular outflow tract; PVL, paravalvular leak; TAVI, transcatheter aortic valve implantation.

**Table 4:** Off-label versus dedicated transcatheter devices in pure native aortic regurgitation.

Feature	Off-Label AS Devices	Dedicated Devices (JenaValve Trilogy; J-Valve)	Current Evidence Level
Anchoring mechanism	Radial force/oversizing against non-calcified annulus	Leaflet-clasping “locators” engaging native cusps	—
Sizing logic	Aggressive annular oversizing: anchoring zone estimated	Device-specific charts based on leaflet capture	—
Principal strength	Immediate availability; operator familiarity	Secure anchoring; low PVL; preserved coronary access	—
Principal failure mode	Migration/embolization; residual AR; second valve	Access-limited; conduction disturbance; cost	—
Best available data	Registries and pooled series (nonrandomized)	ALIGN-AR single-arm pivotal (2-year); meta-analysis	Single-arm/observational; no RCT vs. surgery

Abbreviations: AR, aortic regurgitation; AS, aortic stenosis; PVL, paravalvular leak; RCT, randomized controlled trial.

**Table 5:** Landmark studies underpinning the three complex anatomies.

Study (Anatomy)	Design	N	Devices/Comparator	Follow-Up	Main Takeaway
NOTION-2 (bicuspid/tricuspid) [36]	RCT (low-risk, ≤75 years)	370	TAVI vs. surgery	3 years	Similar overall; caution signal in bicuspid subgroup
Makkar (bicuspid AS) [76]	Propensity-matched registry	2691 pairs	BEV; bicuspid vs. tricuspid	1 year	Comparable death/stroke; more PVL in bicuspid
ALIGN-AR (pure AR) [53]	Single-arm pivotal	180	JenaValve Trilogy (dedicated)	2 years	Met safety/efficacy goals; low PVL; excellent EOA
Meta-analysis dedicated vs. off-label (pure AR) [59]	Meta-analysis (34 studies)	2162	Dedicated vs. off-label	1 year	Lower 30-day mortality and higher success with dedicated
SMART (small annulus) [65]	RCT	716	SEV vs. BEV	2 years	Non-inferior clinically; superior valve performance for SEV
Rheude (small annulus) [67]	Meta-analysis	>7000	BEV vs. SEV	Varies	SEV: larger iEOA, lower gradients, less PPM

Abbreviations: AR, aortic regurgitation; AS, aortic stenosis; BEV, balloon-expandable valve; EOA, effective orifice area; iEOA, indexed EOA; PPM, prosthesis–patient mismatch; PVL, paravalvular leak; RCT, randomized controlled trial; SEV, self-expanding valve.

## Data Availability

No new data were created or analyzed in this study. Data sharing is not applicable to this article.

## Supplementary Materials

The following supporting information can be downloaded at: https://www.mdpi.com/article/10.3390/jcm15197452/s1, Section S1: Literature search and selection; Section S2: Quality appraisal—SANRA checklist; Section S3: Evidence-informed, non-validated framework for Heart Team discussion in complex-anatomy aortic valve disease; Figure S1: Flow of records identified, screened, and retained; Figure S2.1: Flow of literature identification, screening and selection for the anatomy-driven narrative review; Table S1: SANRA adherence checklist for this structured narrative review; Table S2.1: Databases, interfaces, search strings as executed, and records retrieved; Table S2.2: Records identified outside the database search; Table S2.3: Records removed before screening (database branch); Table S2.4. Overlap among databases underlying the 1463 duplicate records removed; Table S2.5: Screening and eligibility counts, by identification route; Table S2.6: Reasons for exclusion of the 229 full-text reports assessed but not retained; Table S2.7: Retained records by study design (n = 101); Table S2.8: Retained records by principal anatomical domain (n = 101); Table S2.9: Retained records by year of publication (n = 101).

## Abbreviations

AR, aortic regurgitation; AS, aortic stenosis; BAV, bicuspid aortic valve; BEV, balloon-expandable valve; CT, computed tomography; EOA, effective orifice area; LVOT, left ventricular outflow tract; PPM, prosthesis–patient mismatch; PVL, paravalvular leak; RCT, randomized controlled trial; SEV, self-expanding valve; STJ, sinotubular junction; THV, transcatheter heart valve; TAVI, transcatheter aortic valve implantation; VARC-3, Valve Academic Research Consortium 3; ViV, valve-in-valve.
