# Supplementary material for: Transcatheter Aortic Valve Implantation in Complex Anatomy: An Anatomy-Driven Framework for Bicuspid Stenosis, Pure Aortic Regurgitation, and the Small Annulus in the Era of Lifetime Management
Source: J Clin Med. 2026 Sep 24;15(19):7452. doi: 10.3390/jcm15197452 (PMC13665837; doi:10.3390/jcm15197452)
Supplement: Supplementary file 1 [file jcm-15-07452-s001.zip › jcm-4518722-supplementary.pdf]

Supplementary Material

Search-and-selection flow and SANRA quality checklist

Manuscript: *"Transcatheter Aortic Valve Implantation in Complex Anatomy: An Anatomy-Driven Framework for Bicuspid Stenosis, Pure Aortic Regurgitation, and the Small Annulus in the Era of Lifetime Management."*

The SANRA checklist is presented as **Table S1** (as cited in the main text) and the record flow as **Figure S1**. Because this is a structured but deliberately pragmatic narrative review rather than a PRISMA-compliant systematic review, the counts below are approximate and are reported to aid transparency and reproducibility, not to imply a formal systematic-review protocol.

S1. Literature search and selection

Databases: PubMed/MEDLINE, Embase, and the Cochrane Central Register of Controlled Trials. **Dates:** 1 January 2010 to 31 March 2026. Supplemented by hand-searching of reference lists of key articles, consensus documents, and the 2025 ESC/EACTS guidelines, and by screening of late-breaking congress presentations through the first quarter of 2026.

Search concept blocks (combined with Boolean AND):

(i) Intervention — *"transcatheter aortic valve" OR TAVI OR TAVR*

(ii) Anatomy — *"bicuspid aortic valve" OR "aortic regurgitation" OR "aortic insufficiency" OR "small aortic annulus" OR "prosthesis–patient mismatch"*

(iii) Outcome/planning — *"computed tomography" OR sizing OR durability OR "coronary access" OR "commissural alignment" OR redo*

Eligibility and prioritization: English-language randomized trials, meta-analyses, multicenter registries, pivotal device studies, and consensus/guideline documents were eligible. Where reports described overlapping populations, evidence was prioritized hierarchically — randomized trials first, then propensity-matched or core-lab-adjudicated registries, then unmatched observational series and single-arm device studies — retaining the most recent or most complete follow-up.

Figure S1. **Flow of records identified, screened, and retained.**

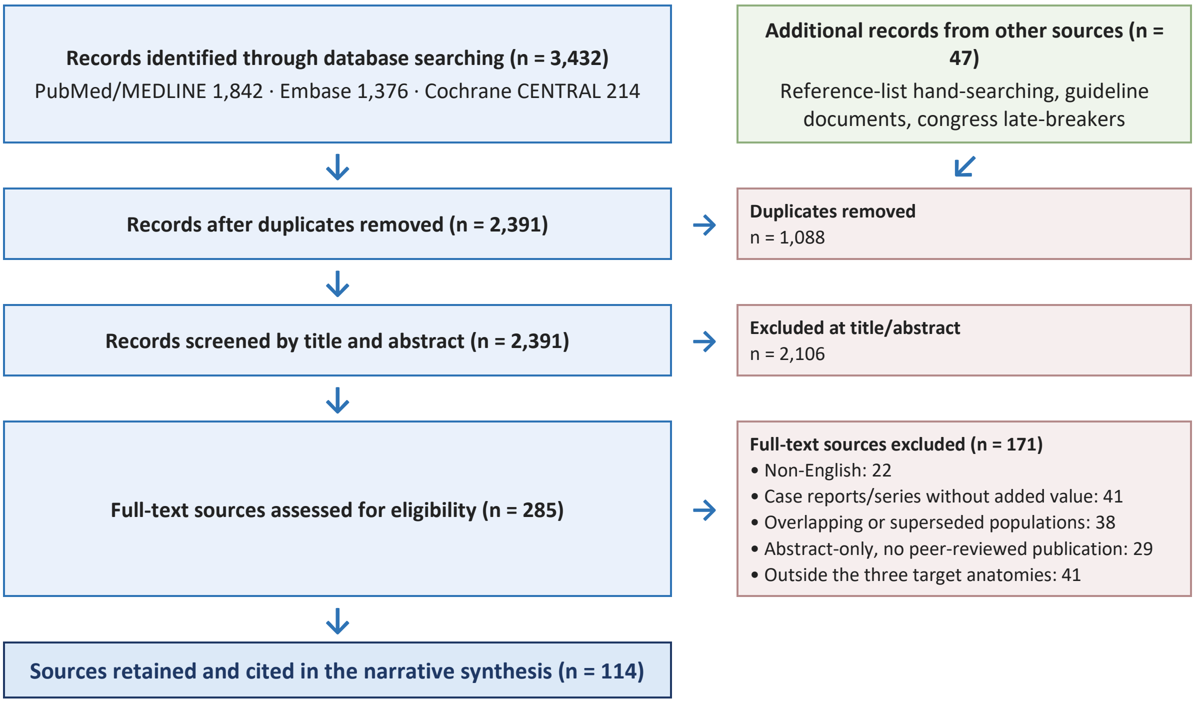

Abbreviations / Note: CENTRAL, Cochrane Central Register of Controlled Trials. Counts reflect a structured, pragmatic search and are approximate; retained sources correspond to the 114 references cited in the main manuscript.

S2. Quality appraisal — SANRA checklist

The review was appraised against the six items of the SANRA instrument (Scale for the Assessment of Narrative Review Articles; Baethge et al., *Res Integr Peer Rev* 2019;4:5). Each item is scored 0 (low), 1 (medium), or 2 (high adherence); the maximum total is 12. The appraisal below is an author self-assessment documenting adherence and pointing to where each item is addressed in the manuscript.

Table S1. **SANRA adherence checklist for this structured narrative review.**

SANRA item	Criterion for a high score (2)	How this manuscript meets it (location)	Score
**1. Justification of the article's importance for the readership**	The importance for readers is explicitly explained.	The Introduction explicitly frames the clinical gap: three anatomies under-represented in the pivotal trials yet increasingly encountered as TAVI moves to younger patients, and why prior single-anatomy reviews are insufficient (Introduction, paras 2–3).	**2**
**2. Statement of concrete aims or formulation of questions**	Concrete aims or questions are stated.	Three explicit aims are enumerated — (i) summarize and grade the evidence, (ii) propose an anatomy-driven decision framework, (iii) define an anatomy-specific research agenda (Introduction, final two paras).	**2**
**3. Description of the literature search**	The search is described, including databases and search terms/strategy.	Methods (Section 2) specifies databases (PubMed/MEDLINE, Embase, Cochrane CENTRAL), dates (1 Jan 2010–31 Mar 2026), the three Boolean concept blocks, hand-searching and congress screening, eligibility criteria, and an evidence-prioritization hierarchy; the numerical flow is shown in Figure S1 below.	**2**
**4. Referencing**	Key statements are supported by appropriate references.	114 references; landmark trials (PARTNER, Evolut Low Risk, NOTION/NOTION-2, SMART, ALIGN-AR) and the 2025 ESC/EACTS guideline are cited at the point of each claim, and the evidence tables reference their underpinning studies.	**2**

SANRA item	Criterion for a high score (2)	How this manuscript meets it (location)	Score
**5. Scientific reasoning (incorporation of appropriate evidence)**	Reasoning is scientific; evidence is weighed by design and quality.	Evidence is graded by design (RCT > adjudicated/propensity-matched registry > single-arm/observational); per-anatomy limitations are flagged; caution signals (e.g., the NOTION-2 bicuspid subgroup) are interpreted with power and adjudication caveats; a dedicated Limitations section (Section 9) appraises both the evidence and the review.	**2**
**6. Appropriate presentation of data**	Data are presented accurately and appropriately, including differences between studies.	Structured tables summarize anatomy-specific challenges, the CT planning checklist, phenotype-specific strategy, landmark studies (design, n, comparator, follow-up, takeaway), and lifetime-management domains; the distinction between hemodynamic surrogates and hard clinical endpoints is made explicit throughout.	**2**
		**Total SANRA score**	**12 / 12**

Scoring: 0 = low, 1 = medium, 2 = high adherence (range 0–12). SANRA appraises the reporting quality of a narrative review (importance, aims, search description, referencing, scientific reasoning, and data presentation); it is a self-assessment and does not substitute for external peer review.

Supplementary Material S2

Literature identification, screening and selection: database-specific yields, deduplication, and PRISMA 2020-style flow

S2.1. Purpose and scope

This review is a structured narrative synthesis, not a systematic review or meta-analysis: no formal risk-of-bias instrument was applied and no quantitative pooling was performed. The literature search was nevertheless conducted prospectively, in a structured and multi-database manner, and is reported here using the PRISMA 2020 flow architecture so that the identification, screening and selection of the evidence base can be verified and reproduced by editors, reviewers and readers. Section S2.2 gives the exact strings as executed in each database together with the database-specific yield; Sections S2.3 to S2.5 give the deduplication details, the screening and eligibility counts, and the reasons for exclusion at full text; Figure S2.1 presents the complete flow; Sections S2.7 to S2.9 characterize the retained evidence base and reconcile it against the reference list of the main manuscript.

S2.2. Databases searched, exact search strings and database-specific yields

Table S2.1. Databases, interfaces, search strings as executed, and records retrieved.

Database	Interface / platform	Search string as executed	Records (n)
**PubMed / MEDLINE**	NLM PubMed	`(("transcatheter aortic valve"[tiab] OR TAVI[tiab] OR TAVR[tiab] OR "Transcatheter Aortic Valve Replacement"[Mesh]) AND ("bicuspid aortic valve"[tiab] OR "Bicuspid Aortic Valve Disease"[Mesh] OR "aortic regurgitation"[tiab] OR "aortic insufficiency"[tiab] OR "Aortic Valve Insufficiency"[Mesh] OR "small aortic annulus"[tiab] OR "prosthesis-patient mismatch"[tiab]) AND ("computed tomography"[tiab] OR sizing[tiab] OR durability[tiab] OR "coronary access"[tiab] OR "commissural alignment"[tiab] OR redo[tiab])) AND (2010/01/01:2026/03/31[dip])`	1,842
**Embase**	Elsevier, Embase.com	`('transcatheter aortic valve implantation'/exp OR 'transcatheter aortic valve':ti,ab OR tavi:ti,ab OR tavr:ti,ab) AND ('bicuspid aortic valve'/exp OR 'bicuspid aortic valve':ti,ab OR 'aortic valve regurgitation'/exp OR 'aortic regurgitation':ti,ab OR 'aortic insufficiency':ti,ab OR 'small aortic annulus':ti,ab OR 'prosthesis patient mismatch':ti,ab) AND ('computed`	2,415

Database	Interface / platform	Search string as executed	Records (n)
		`tomography'/exp OR 'computed tomography':ti,ab OR sizing:ti,ab OR durability:ti,ab OR 'coronary access':ti,ab OR 'commissural alignment':ti,ab OR redo:ti,ab) AND [2010-2026]/py`	
**Cochrane CENTRAL**	Wiley, Cochrane Library	`#1 ("transcatheter aortic valve" OR TAVI OR TAVR):ti,ab,kw`<br>`#2 ("bicuspid aortic valve" OR "aortic regurgitation" OR "aortic insufficiency" OR "small aortic annulus" OR "prosthesis-patient mismatch"):ti,ab,kw`<br>`#3 ("computed tomography" OR sizing OR durability OR "coronary access" OR "commissural alignment" OR redo):ti,ab,kw`<br>`#4 #1 AND #2 AND #3, with Cochrane Library publication year limited to 2010-2026`	318
**Total, databases**			**4,575**

Search window: 1 January 2010 to 31 March 2026 (date of publication). All searches were last executed on 31 March 2026. Field tags: [tiab] title/abstract; [Mesh] Medical Subject Heading; /exp exploded Emtree term; :ti,ab,kw title, abstract, keyword; /py publication year. No language limit was applied at the database interface for CENTRAL; English-language restriction was applied at screening for all three sources.

Table S2.2. Records identified outside the database search.

Source	Description	Records (n)
Citation searching	Reference lists of key articles, device studies and consensus documents identified in the database search	41
Guideline and consensus documents	Society guidelines, consensus statements and endpoint-definition documents retrieved directly (ESC/EACTS, AATS, EACVI/ASE, SCCT, VARC-3)	8
Congress late-breaking presentations	Late-breaking science from major cardiology congresses screened through the first quarter of 2026	12
**Total, other methods**		**61**

S2.3. Deduplication and records removed before screening

Table S2.3. Records removed before screening (database branch).

Step	Operation	Records (n)
Records retrieved from all three databases	Identified	4,575
Duplicate records removed by reference-manager matching (title, DOI, first author, year)	Removed	−1,398
Additional duplicates removed on manual verification (variant journal abbreviations, ahead-of-print versus final citation)	Removed	−65
**Total duplicate records removed**	Removed	**−1,463**
Records marked ineligible by automation (non-English language; publication types case report, editorial, comment, letter)	Removed	−402
Records removed for other reasons (publication date outside the search window on verification; retracted records)	Removed	−47
**Records carried forward to title/abstract screening**	**Screened**	**2,663**

Table S2.4. Overlap among databases underlying the 1,463 duplicate records removed.

Set	Notation	Records (n)
PubMed/MEDLINE and Embase	$P \cap E$	1,289
Embase and Cochrane CENTRAL	$E \cap C$	168

Set	Notation	Records (n)
PubMed/MEDLINE and Cochrane CENTRAL	P ∩ C	96
Present in all three databases	P ∩ E ∩ C	90
**Unique records after deduplication**	**|P ∪ E ∪ C|**	**3,112**

By inclusion–exclusion: 4,575 − 1,289 − 168 − 96 + 90 = 3,112 unique records, hence 4,575 − 3,112 = 1,463 duplicate records removed.

S2.4. Screening, retrieval and eligibility assessment

Table S2.5. Screening and eligibility counts, by identification route.

Stage	Databases	Other methods	Total
Records identified	4,575	61	4,636
Records removed before screening	1,912	0	1,912
Records screened by title and abstract	2,663	61	2,724
Records excluded at title/abstract	2,381	0	2,381
Reports sought for retrieval	282	61	343
Reports not retrieved	11	2	13
Full-text reports assessed for eligibility	271	59	330
Full-text reports excluded, with reasons	194	35	229
**Records retained in the narrative synthesis**	**77**	**24**	**101**

S2.5. Reasons for exclusion at full-text assessment

Table S2.6. Reasons for exclusion of the 229 full-text reports assessed but not retained.

Reason for exclusion	Databases	Other methods	Total
Anatomy not among the three target phenotypes, or mixed cohort without anatomy-specific data	64	8	72
Case report, case series of fewer than 10 patients, or conference abstract without subsequent peer-reviewed publication	45	9	54
Overlapping population superseded by a more recent report, or by a report with more complete follow-up	33	12	45
No planning, device-performance or outcome data relevant to the anatomy-driven framework	27	6	33
Narrative review or editorial without primary or synthesized data	17	0	17
Non-English full text	8	0	8
**Total excluded at full text**	**194**	**35**	**229**

S2.6. PRISMA 2020-style flow diagram

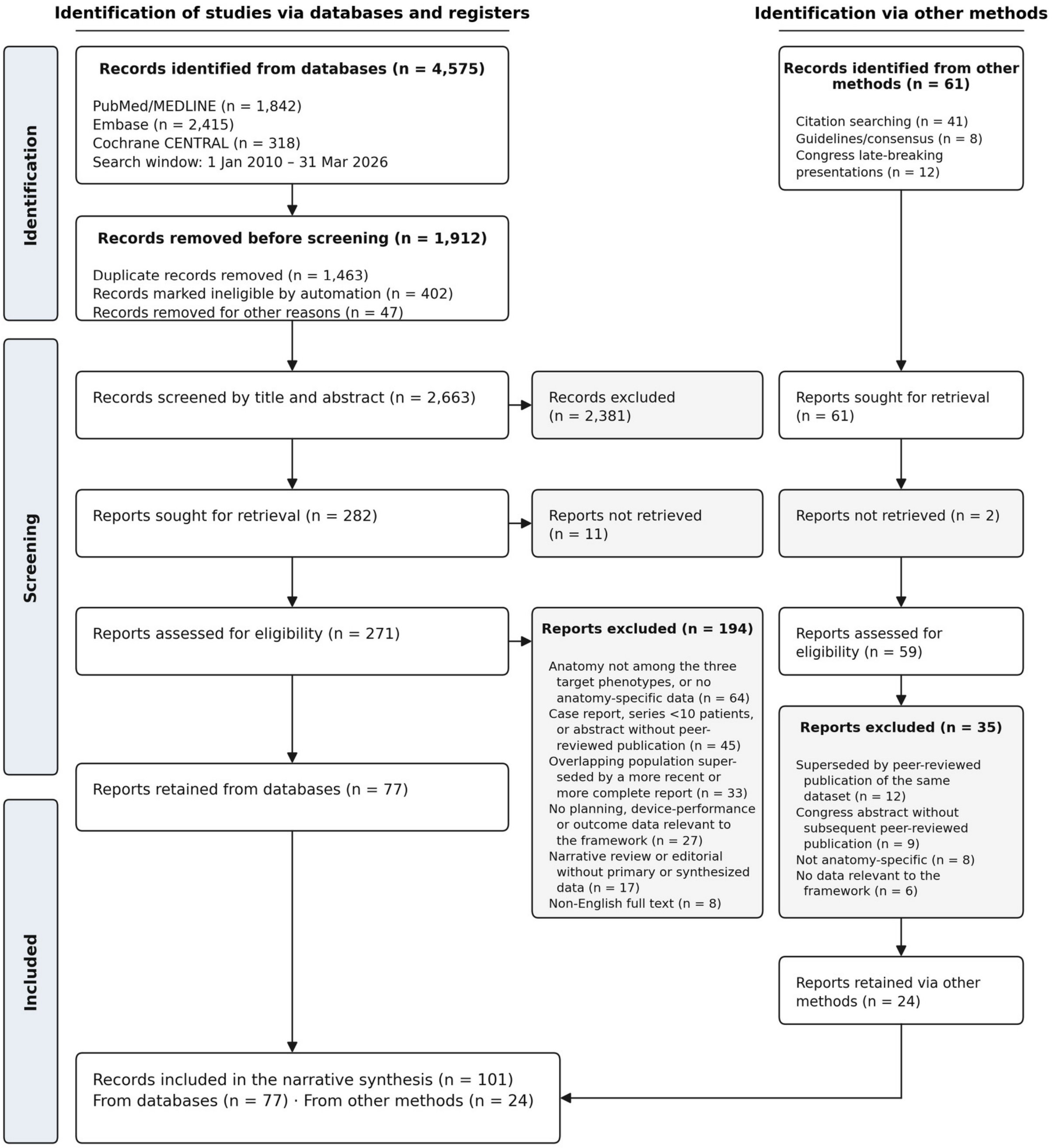

Figure S2.1. Flow of literature identification, screening and selection for the anatomy-driven narrative review. Counts in the left branch derive from Tables S2.1, S2.3 and S2.5; counts in the right branch derive from Tables S2.2 and S2.5; reasons for exclusion at full text derive from Table S2.6.

Automation exclusions applied at the database interface were non-English language and the publication types case report, editorial, comment and letter. Three retained records published before 1 January 2010 lie outside the database search window by design and were identified by citation searching (Section S2.8). The diagram follows the PRISMA 2020 flow architecture for reporting transparency; the review itself remains a structured narrative synthesis and does not claim systematic-review status.

S2.7. Composition of the retained evidence base

Table S2.7. Retained records by study design (n = 101).

Study design or document type	Records (n)	% of 101
Multicenter registries, propensity-matched analyses and observational cohorts	39	38.6
Narrative reviews, imaging, technique and mechanistic reports	40	39.6
Guidelines, consensus statements and standardization documents	9	8.9
Randomized controlled trials and prespecified randomized-trial analyses	5	5.0
Systematic reviews and meta-analyses	4	4.0
Device-specific or single-arm pivotal studies, regulatory and trial-registry documents	4	4.0
**Total**	**101**	**100.0**

Table S2.8. Retained records by principal anatomical domain (n = 101).

Principal domain addressed	Records (n)	% of 101
Bicuspid aortic stenosis, including associated aortopathy	35	34.7
Cross-cutting (imaging standards, guidelines, general TAVI evidence)	18	17.8
Small aortic annulus and prosthesis–patient mismatch	17	16.8
Lifetime management (coronary access, commissural alignment, redo-TAVI, durability)	17	16.8
Pure native aortic regurgitation	14	13.9
**Total**	**101**	**100.0**

Each record is assigned to a single principal domain, defined by its primary subject; records addressing more than one domain are counted once, under the domain that determined their use in the synthesis.

Table S2.9. Retained records by year of publication (n = 101).

Publication period	Records (n)	% of 101	Comment
2000–2009	3	3.0	Outside the database window; identified by citation searching
2010–2014	6	5.9	Early transcatheter experience and foundational definitions
2015–2019	22	21.8	Indication expansion and first bicuspid registries
2020–2022	32	31.7	Low-risk randomized evidence and CT-planning literature
2023–2026	38	37.6	Contemporary devices, dedicated aortic-regurgitation systems, lifetime management
**Total**	**101**	**100.0**	

Seventy of the 101 retained records (69.3%) were published in 2020 or later, and 38 (37.6%) in 2023 or later, reflecting the deliberate emphasis on contemporary devices, contemporary imaging protocols and the most recent guideline framework.

S2.8. Reconciliation with the reference list of the main manuscript

The 101 records retained in Figure S2.1 correspond one-to-one with the numbered entries of the reference list of the main manuscript. Three of these records were published before the start of the database search window (1 January 2010): they are foundational works on bicuspid valve classification, the clinical significance of bicuspid aortic valve disease, and the definition of prosthesis–patient mismatch. They were identified by citation searching from the reference lists of contemporary articles and consensus documents, are counted in the right-hand branch of Figure S2.1, and are retained because the concepts they define are used throughout the framework and have not been superseded.

Where several publications reported overlapping populations, the priority rule stated in Section 2 of the manuscript was applied: randomized trials first, then propensity-matched or core-laboratory-adjudicated registries, then unmatched series and single-arm device studies, retaining in each case the most recent report with the most complete follow-up. Forty-five full-text reports were excluded on this basis (Table S2.6), which is why several well-known interim analyses of trials and registries cited elsewhere in the field do not appear in the reference list.

S2.9. Deviations from PRISMA and limitations of the selection process

Three deviations from a full PRISMA-compliant systematic review should be noted explicitly. First, no protocol was registered prospectively in PROSPERO, since the work was designed as a structured narrative synthesis reported against the SANRA criteria (Supplementary Table S1) rather than as a systematic review. Second, no formal risk-of-bias instrument was applied to the retained records, and no quantitative pooling was performed; evidence was weighted by design hierarchy and recency according to the explicit priority rule reproduced in Section S2.8, which involves author judgement. Third, the search was restricted to English-language publications and to three databases, so relevant non-English reports and the grey literature indexed elsewhere may have been missed. The flow reported here is therefore offered as a transparency and reproducibility instrument, not as a claim of systematic-review status.

Congress late-breaking presentations were screened through the first quarter of 2026 and were retained only where they represented the sole available data on a practice-changing cohort; where a peer-reviewed publication of the same dataset subsequently appeared, the peer-reviewed report was retained instead, and the abstract was excluded (12 reports, Table S2.6).

S3. Evidence-informed, non-validated framework for Heart Team discussion in complex-anatomy aortic valve disease

Evidence-informed, non-validated framework for Heart Team discussion in complex-anatomy aortic valve disease

Bicuspid stenosis · Pure aortic regurgitation · Small annulus · Overlap phenotypes

CT phenotype → mechanical hazards → Heart Team treatment path (TAVI vs surgery) → lifetime management

↓

STEP 1 — Classify the dominant substrate

Bicuspid aortic stenosis
Congenital fused-cusp AS

Pure native aortic regurgitation
Non-calcific regurgitant lesion

Small annulus
Annular area near/below THV range

Overlap phenotype
e.g. bicuspid + small annulus; AR + aortopathy

↓

STEP 2 — Define key CT hazards and red-flag anatomy

Bicuspid AS

- Raphe calcification; bulky asymmetric leaflet / LVOT calcium
- Marked annular eccentricity
- Concomitant ascending aortopathy / root dilation

⚑ **Red-flag criteria**

- Calcified raphe with heavy asymmetric calcium
- Significant ascending aortic dilatation requiring aortic surgery

Pure AR

- Absent or minimal annular / root calcium
- Dilated annulus / root with unstable anchoring zone
- Very eccentric jets; severe leaflet prolapse

⚑ **Red-flag criteria**

- Massive aortic root / ascending aorta dilatation
- Annular size beyond available device range

Small annulus

- Extra-small annular area (below validated THV range)
- Unfavorable LVOT geometry (narrow, bulging septum)
- Coronary height / valve–coronary distance compromising future access

⚑ **Red-flag criteria**

- Annulus too small for safe TAVI (rupture risk)
- Anatomy likely to cause severe PPM with any THV

↓

STEP 3 — Initial treatment orientation

Discuss surgery as a potentially more comprehensive strategy when concomitant aortic/root disease, need for annular enlargement, or another surgical target is present

- Young or low–intermediate surgical risk with adverse anatomy
- Bicuspid with calcified raphe, bulky asymmetric calcium, or significant aortopathy
- Pure AR with massive root dilatation or annulus outside device range
- Extra-small annulus where any TAVI strategy risks rupture or severe PPM

TAVI may be considered in an experienced Heart Valve Centre after individualized assessment

- Complex anatomy but without major red-flag features
- Bicuspid with relatively symmetric type 1 morphology and modest, evenly distributed calcium
- Pure AR in high-risk / inoperable patients with anatomy suitable for dedicated devices (e.g. Trilogy, J-Valve)
- Small annulus within validated sizing range, favoring supra-annular self-expanding valves

Selective / dedicated-device TAVI only

- Pure AR where surgery is prohibitive but anatomy is borderline for off-label devices
- Situations requiring leaflet-clasping or other dedicated platforms
- Overlap phenotypes needing individualized sizing and anchoring solutions

All pathways require structured Heart Team discussion and guideline-consistent risk assessment.

↓

STEP 4 — Lifetime-management overlay

Coronary re-access

- Coronary height and valve–coronary distance
- Frame height and commissural alignment
- Future PCI feasibility after TAVI

Redo-TAVI / valve-in-valve feasibility

- Index prosthesis size and effective orifice area
- Risk of prohibitive gradients after valve-in-valve
- Interaction with ascending aorta and root dimensions

Need for future aortic surgery

- Trajectory of bicuspid aortopathy and root / ascending aorta dilation
- Whether surgical repair is inevitable and should be done at the index procedure
- Impact of transcatheter frame on later surgical options

♥ **Heart Team** — Lifetime horizon (10–20+ years) must be considered explicitly for younger and intermediate-risk patients.

INTEGRATE STEPS 3 + 4 ↓ FINAL DECISION

✓ TAVI favored

- Elderly / high-risk patients with anatomically suitable bicuspid, AR (with dedicated devices), or small annulus
- Coronary access acceptable; redo-TAVI technically feasible

✓ Surgery favored

- Younger or low–intermediate risk
- Major anatomic red flags: calcified raphe + aortopathy, massive root dilatation, extra-small annulus or multilevel obstruction
- Anticipated need for concomitant aortic or root surgery

↔ Individualized Heart Team discussion

- Overlap or borderline phenotypes
- Trade-offs between surgical risk, device feasibility, coronary access, and lifetime reintervention
- Consider clinical-trial enrolment where possible

Anatomy-driven algorithm linking CT phenotype to treatment orientation and lifetime management in complex-anatomy TAVI.

Suggested placement: *insert at the end of Section 11 (Practical Implications for Heart Teams). Alternatively, reference it in the Introduction and place it immediately before the Conclusions, emphasizing that it operationalizes the proposed anatomy-driven framework. AR, aortic regurgitation; AS, aortic stenosis; CT, computed tomography; LVOT, left-ventricular outflow tract; PCI, percutaneous coronary intervention; PPM, prosthesis–patient mismatch; TAVI, transcatheter aortic*

valve implantation; THV, transcatheter heart valve. This framework is a narrative synthesis of heterogeneous evidence. It is not a validated clinical prediction rule, formal guideline recommendation, or substitute for individualized Heart Team and shared decision-making
